# GABenchToB: A Genome Assembly Benchmark Tuned on Bacteria and Benchtop Sequencers

**DOI:** 10.1371/journal.pone.0107014

**Published:** 2014-09-08

**Authors:** Sebastian Jünemann, Karola Prior, Andreas Albersmeier, Stefan Albaum, Jörn Kalinowski, Alexander Goesmann, Jens Stoye, Dag Harmsen

**Affiliations:** 1 Department for Periodontology, University of Münster, Münster, Germany; 2 Institute for Bioinformatics, Center for Biotechnology, Bielefeld University, Bielefeld, Germany; 3 Technology Platform Genomics, Center for Biotechnology, Bielefeld University, Bielefeld, Germany; 4 Bioinformatics Resource Facility, Center for Biotechnology, Bielefeld University, Bielefeld, Germany; 5 Bioinformatics and Systems Biology, Justus-Liebig-Univeristy Gießen, Gießen, Germany; 6 Genome Informatics Group, Faculty of Technology, Bielefeld University, Bielefeld, Germany; CNRS UMR7622 & University Paris 6 Pierre-et-Marie-Curie, France

## Abstract

*De novo* genome assembly is the process of reconstructing a complete genomic sequence from countless small sequencing reads. Due to the complexity of this task, numerous genome assemblers have been developed to cope with different requirements and the different kinds of data provided by sequencers within the fast evolving field of next-generation sequencing technologies. In particular, the recently introduced generation of benchtop sequencers, like Illumina's MiSeq and Ion Torrent's Personal Genome Machine (PGM), popularized the easy, fast, and cheap sequencing of bacterial organisms to a broad range of academic and clinical institutions. With a strong pragmatic focus, here, we give a novel insight into the line of assembly evaluation surveys as we benchmark popular *de novo* genome assemblers based on bacterial data generated by benchtop sequencers. Therefore, single-library assemblies were generated, assembled, and compared to each other by metrics describing assembly contiguity and accuracy, and also by practice-oriented criteria as for instance computing time. In addition, we extensively analyzed the effect of the depth of coverage on the genome assemblies within reasonable ranges and the k-mer optimization problem of de Bruijn Graph assemblers. Our results show that, although both MiSeq and PGM allow for good genome assemblies, they require different approaches. They not only pair with different assembler types, but also affect assemblies differently regarding the depth of coverage where oversampling can become problematic. Assemblies vary greatly with respect to contiguity and accuracy but also by the requirement on the computing power. Consequently, no assembler can be rated best for all preconditions. Instead, the given kind of data, the demands on assembly quality, and the available computing infrastructure determines which assembler suits best. The data sets, scripts and all additional information needed to replicate our results are freely available at ftp://ftp.cebitec.uni-bielefeld.de/pub/GABenchToB.

## Introduction

With the introduction of massively parallel high-throughput next generation sequencing (NGS) platforms, fast and cost-effective whole genome shotgun sequencing of the full variety of organisms has been enabled. The rapid advancement in this field is best represented by the recently introduced small scaled benchtop sequencers (BS), e.g., the MiSeq by Illumina (San Diego, California) and the Ion Torrent PGM by Life Technologies (Carlsbad, California). Albeit providing a lower throughput than their conventional non-benchtop counterparts (e.g., Illumina's HiSeq and Ion Torrent's Proton), they still provide sufficient genomic coverage and sequencing accuracy to be efficiently used for sequencing bacterial genomes [1]–[2].

One crucial step in genome based analysis is the attempt to *de novo* assemble raw sequencing reads into a bacterial chromosome. *De novo* genome assembly is the process of reconstructing a whole genome sequence from short sequencing reads by finding common subsequences and assembling overlapping reads to longer continuous sequences, i.e. contigs, under the assumption that such reads originate from the same genomic location. If special pairs of reads with a known pairing distance are available, i.e. mate-pair (MP) or paired-end (PE) reads, this information can be used to arrange individual contigs in an ordered sequence consisting of contigs and gaps of known sizes (scaffolds). This is in particular useful to span gaps related to long repetitive elements which are hard to be resolved solely by overlapping reads of limited length. In general, most current assembly algorithms can be assigned to one of two classes based on their underlying data structure: de Bruijn graph (DBG) and overlap layout consensus (OLC) assemblers. Both approaches utilize a graph structure built upon the sequencing reads and algorithms for graph traversal in order to deduce overlapping sequences and to generate contigs. Very briefly, OLC assemblers build a graph by connecting nodes which represent the sequencing reads by edges representing the specific overlaps. For the DBG approach reads are initially partitioned into substrings of the reads of a fixed length (k-mers) and a graph is built by connecting nodes symbolizing sub-reads that share a specific prefix and suffix, respectively. See Compeau et al. 2011 [3], Li et al. 2012 [4] and Nagarajan and Pop 2013 [5] for detailed descriptions on the principles of DBG and OLC assemblers.

The rapid progress in the field of NGS as well as diverse sequencing procedures and protocols had also impacts on genome assembly algorithms and assembly software solutions. The number of sequence assemblers steadily increased in recent years with currently several dozens of assemblers available (22 genome assemblers compared in 2008 [6], additional 13 assemblers in 2010 [7], further ten assemblers in 2011 [8]). Although this variety becomes somewhat more limited as some assemblers require a particular kind or set of sequencing data as an input, researchers are still confronted with a wide range of assembler candidates. The decision which *de novo* assembler to use is conditional to several aspects, notably the specification of the applied sequencing platform and protocol (e.g., single-end versus PE reads), characteristics of the sequencing results (read lengths and error profiles) and, if available, characteristics of the sequenced genome (e.g., number of repetitive genomic elements or the genomic %GC-content).

Therefore, systematic evaluations of assemblers are necessary to provide the research community with scientifically sound decision-making support. In the past, several efforts have been made to assess genome assembler efficiency on different scales and for different application scenarios [8]–[11]. Most prominently, two comparative studies with recently introduced remakes contributed greatly to this field, i.e. Assemblathon and Genome Assembly Gold-Standard Evaluations (GAGE). For the first large scale assembler competition Assemblathon 1 [12] and its recent successor Assemblathon 2 [13] assemblies were performed and submitted by different institutions and thereafter evaluated. In the first study, 17 participants generated 41 assemblies based on simulated short Illumina HiSeq reads covering 16 different genome assemblers. In Assemblathon 2, 21 participants assembled 43 genomes of three vertebrate species sequenced on different instruments while using eight genome assemblers. In contrast, for the GAGE [14] and GAGE-B [15] competitions all assemblies were generated under equal conditions. Whereas GAGE compared eight different genome assemblers using multiple Illumina libraries of four different pro- and eukaryotic data sets, GAGE-B concentrated on single library assemblies of nine Illumina sequenced bacterial strains using also eight different assemblers.

Albeit these studies give a very comprehensive picture of the efficiency and applicability of state-of-the-art assembler algorithms, several questions remain unanswered. First and foremost, NGS is evolving fast and consumables, protocols, and technical specifications of BS differ in comparison to conventional NGS instruments. For instance, the MiSeq offers an improved read length of 2×300 base pairs (bp) compared to the maximum 2×150 bp of the HiSeq system. The Ion Torrent PGM, although comparable to Roche's 454 in terms of the error profile, produces a different read length distribution (current maximum read length of about 400 bp for the PGM compared to the 1,000 bp at max. for the 454 GS−FLX+ system). Up to now, only the GAGE-B evaluation took into account MiSeq data sets while evaluations of assemblies originating from PGM instruments are missing. Therefore, methods need to be reconsidered for deciding how to assemble data originating from BS instruments. Secondly, bacterial genomes are underrepresented, particularly in the Assemblathon surveys. Even if bacterial genomes are lightweights regarding their assembly difficulty compared to e.g. vertebrates, they nevertheless have different requirements on sequencing procedures and the assembler algorithms. GAGE-B recently tried to close this gap, yet for the majority of the used data sets a high quality genome reference was not available. A third aspect of an assembler evaluation is more of a practice-oriented nature, e.g. the run time and memory usage of an assembly and the demands each assembler has on the compute infrastructure. An assembler recommendation would have little practical value if best performing assemblers cannot be operated due to impractical hardware requirements. However, this aspect was covered insufficiently in the past. Finally, a comparison study should be transparent in order to sustain reproducibility. This means that all steps beginning from the raw sequencing data to the final results should be sufficiently documented. Especially the Assemblathon competitions could not fully satisfy this requirement owed to the fact that the assemblies were performed at different institutions and documented at varying extent.

Here, we present a *de novo* assembler evaluation with a strong focus on practical aspects and which addresses the aforementioned unanswered questions. To this end, the main objectives of this Genome Assembly Benchmark Tuned On Bacteria and benchtop sequencers (GABenchToB) are:

- to use real (no synthetic) data originating from benchtop sequencers,
- to use single libraries only of bacterial genomes with an available high-quality reference,
- to consider open licensed as well as commercial assemblers,
- to select assemblers covering different assembly strategies,
- to not perform extensive assembly fine tuning but to rest on default parameters for the different sequencing platforms where possible and to use unprocessed raw reads,
- to include run time benchmark parameters, and
- to ensure for equal executing conditions by using dedicated computing hosts and to deposit all information necessary for reproduction at an open repository.

## Results

### Evaluation data sets

We have chosen three different bacterial strains for our assembly evaluation: *Escherichia coli O157:H7 Sakai* (American Tissue Culture Collection [ATCC] accession no. BAA-460), *Staphylococcus aureus COL* (Network on Antimicrobial Resistance in *Staphylococcus aureus* [NARSA] accession no. NRS100), and *Mycobacterium tuberculosis H37Rv* (ATCC accession no. 25618). For all three bacteria full reference sequences are available: NCBI Reference Sequence (RefSeq) accession no. NC_002695.1, NC_002128.1 and NC_002127.1 with modifications as described previously [2] were used as a reference for the *E. coli* genome and plasmids; NC_002951.2 and NC_006629.2 for the *S. aureus* genome and plasmid, and NC_000962.2 for the *M. tuberculosis* genome, respectively. The genomes cover a GC-content between 33 and 66% and have a genome size between 2.81 and 5.59 megabases (Mb). Samples of the three bacterial strains were sequenced on BS platforms Ion Torrent PGM (PGM) by Life Technologies and MiSeq (MIS) by Illumina. Both platforms are not only represented by all three organisms but also with different chemistries, i.e. 2×150 base pairs (bp) PE and 2×250 bp PE sequencing for the MiSeq as well as 200 bp and 400 bp sequencing for the PGM, resulting in a total of ten single library data sets (**Table 1**). Except for the four *E. coli* data sets, which were used and published previously [2], all other libraries were generated newly for the purpose of this study.

**Table 1:** Overview of the data sets used in this study and their sequencing yield.

Platform	Library	Software version	Strain	Chip/Lane	Megabases	Coverage
PGM	200 bp& Ion Xpress Plus Fragment**	TSS v3.0$	*E. coli* (Sakai)	1×316 chip	733	133
PGM	400 bp Ion Xpress Plus Fragment**	TSS v3.4$	*E. coli* (Sakai)	1×318 chip	1,179	214
PGM	200 bp Ion Xpress Plus Fragment	TSS v3.0$	*S. aureus* (COL)	1×316 chip	555	197
PGM	400 bp Ion Xpress Plus Fragment	TSS v3.2$	*S. aureus* (COL)	1×318 chip	1,420	505
PGM	400 bp Ion Xpress Plus Fragment	TSS v3.6$	*M. tuberculosis* (H37)	1/3 x318 chip	344	77
MiSeq	2×150 bp PE+ Nextera*	MCS v1.2.3	*E. coli* (Sakai)	¼ multiplexed lane	565	102
MiSeq	2×250 bp PE Nextera*	MCS v2.0.5	*E. coli* (Sakai)	¼ multiplexed lane	776	141
MiSeq	2×150 bp PE Nextera	MCS v1.2.3	*S. aureus* (COL)	¼ multiplexed lane	445	158
MiSeq	2×250 bp PE Nextera	MCS v2.0.5	*S. aureus* (COL)	¼ multiplexed lane	509	181
MiSeq	2×250 bp PE Nextera	MCS v2.0.5	*M. tuberculosis* (H37)	15% multiplexed lane	340	77

& base pairs.

+ Paired-end sequencing.

*Same raw data set as in Jünemann et al. [2].

**Same raw data set as in Jünemann et al. [2] but re-analyzed using a different sequencing software version. MiSeq, Illumina MiSeq; PGM, Ion Torrent Personal Genome Machine. MCS, MiSeq Control Software; TSS, PGM Torrent Suite Software.

$ More stringent filter enabled.

### *De novo* assembler selection

To enable an assembler evaluation we aimed to select a set of *de novo* assemblers regarding the following criteria: assemblers which are (i) representing DBG or OLC approaches; (ii) free-to-use or open source as well as commercial products; (iii) unbiased in terms of the supported sequencing technology or the required sequencing library (processing single and paired-end reads, not relying on mate-pair libraries and no requirement on multiple libraries); (iv) up-to-date regarding to the date of their release or the latest software update; and (v) established and widely used either in recent *de novo* sequencing projects or in other assembler evaluations. Based on these criteria we selected nine representative assemblers: AbySS [16], Celera [17], CLC Assembly Cell (CLC bio A/S, Aarhus, Denmark) [18], GS De Novo Assembler (454 Life Sciences, Branford, CT) [19], MIRA [20], SeqMan Ngen (DNASTAR Inc., WI, USA) [21], SOAPdenovo2 [22], SPAdes [23], and Velvet [24] (**Table 2**). Some assemblers we did not include in our study, but which performed best in either one or several categories in previous evaluations [11]–[15] are: Allpaths-LG [25] because its algorithm requires input data consisting of at least two different libraries (one PE and one MP), ARACHNE [26] because it combines only long Sanger with MP reads, MaSuRCA [27] and SGA [28] because both can not handle single-end data, and Phusion [29] because it builds solely upon MP reads.

**Table 2:** *De novo* assemblers used for comparison.

Assembler (acronym)	Software version	Type	Supports scaffolding	License	Supported operating systems
AbySS (ABYSS)	1.3.5	DBG$	yes	commercial*	Windows, Linux
Celera (CELERA)	7.0	OLC&	yes	open source	Windows, Linux
CLC Assembly Cell (CLC)	4.0.10	DBG	yes	commercial	Windows, Linux, Mac OS
GS De Novo Assembler (NEWBLER)	2.8	OLC	yes	commercial**	Linux
MIRA (MIRA)	3.9.9	OLC#	no	open source	Linux
SeqMan Ngen (SEQMAN)	11.0.0.172	OLC	no	commercial	Windows, Linux, Mac OS
SOAPdenovo2 (SOAP2)	2.04	DBG	yes	open source	Linux
SPAdes (SPADES)	2.5.0	DBG	yes	open source	Linux, Mac OS
Velvet (VELVET)	1.2.08	DBG	yes	open source	Linux, Mac OS

$ de Bruijn Graph assembler,

& Overlap Layout Consensus assembler,

# MIRA is not a pure OLC assembler but uses also greedy assembler techniques.

^*^Free for non-commercial and academic applications.

^**^Freely available upon request.

### Assembly evaluation metrics

The evaluation of genome assemblers is a complex problem and single metric based evaluations using e.g., the N50 or NG50 value for comparisons examine only specific aspects of an assembly [9]. On the other hand, Haiminen et al. [9] criticize that providing tables full of different assembly metrics complicate the understanding and interpretation of an evaluation effort and limit their usability in practice. A single metric capturing the trade-off between contig contiguity and accuracy are feature response curves (FRC) as proposed by Narzisi and Mishra [11]. However, one limitation of FRC is their requirement of read based contig layouts, which are naturally not available for DBG assemblers. To overcome this limitation, Haiminen et al. [9] introduced FRCbam, which allows generating a read layout by aligning the sequencing reads to the assembled contigs by incorporating paired-end and mate-pair information. However, this is also a strong limitation for the evaluation of assemblies based on BS data, as no paired-end libraries are available (PGM) or are of limited use due to their small insert-size (MiSeq). Another single metric is the Log Average probability proposed by Ghodsi et al. [30] but it was designed to measure assemblies without knowledge of the true reference. Here, we assessed assembly accuracy using the recently published assembly evaluation software QUAST [31] following in essence the GAGE-B evaluation [15]. For that matter, assembly contiguity and accuracy are measured by two metrics introduced by QUAST and another two metrics are used to measure system workload: i.e. the NGA50 length, the number of mis-assemblies, the total run time (wall clock time), and the average system utilization (see the Methods section for a detailed definition of the metrics).

It is to be noted that we did not compare assemblies and platforms separately for contigs and scaffolds in order to preserve a focused evaluation. From a pragmatic point of view it is contradictory to use contigs for an evaluation even though scaffolds of the same assembly are available. Additionally, scaffolding requires PE or MP reads. As no PE-libraries have been available for the PGM platform scaffolding of PGM data was not possible but disabling scaffolding would unnecessarily disfavor the MiSeq data sets. Therefore, the term contig is used, with appropriate remarks, ambiguously for both contigs and scaffolds (see **Table 1** for an overview which data sets are based on PE data and **Table 2** which assembler supports scaffolding).

### Genome coverage

One major parameter that influences the result of a genome assembly is the amount of available data to cover the whole genome, i.e. the depth of coverage. Insufficient coverage may lead to an inferior assembly result either due to uncovered genomic regions or due to the impossibility to overrule randomly distributed sequencing errors. In contrast, each increase of the target coverage involves a higher load of the sequencing platform capacity, which comes at the price of an increased sequencing cost per library as well as of a growing computational effort due to the increased data volume. Also, non-randomly distributed sequencing errors (e.g., systematic errors related to homopolymer regions) can not be ruled out by redundant sequencing and accumulate with increasing coverage which can trick assemblers to handle them as true biological events [32]. Therefore, finding a balance between sequencing effort, cost, assembly turn-around time and assembly quality is crucial in advance to the sequencing itself. The observation that an ad infinitum increased depth of coverage does not necessarily improve assembly results was already reported by Lin et al. [10]. They found that for seven assemblers operated on six simulated data sets based on a eukaryotic chromosome the depth of coverage at which the N50 length plateau was reached never exceeded values of 50. Consequentially, an upper coverage threshold of 70x was used to compare the assembly results. Similar examinations in further studies yielded comparable results [9], [15], [33]. However, common to all these studies were shortcomings regarding the resolution, the range of assemblers, the amount of data at which this effect was studied, and most of all only Illumina reads were used. To address the question to what extent depth of coverage affects assembly results we have randomly sub-sampled all PGM and MiSeq data sets into a fix range of subsets. All subsets were subsequently assembled using three DBG (CLC, ABYSS, and SPADES) and three OLC assemblers (NEWBLER, CELRA, and MIRA). This reduced assembler set was chosen to cope with the high computational effort while maintaining meaningful results with regard to our assembler selection criteria.

**Figure 1:**
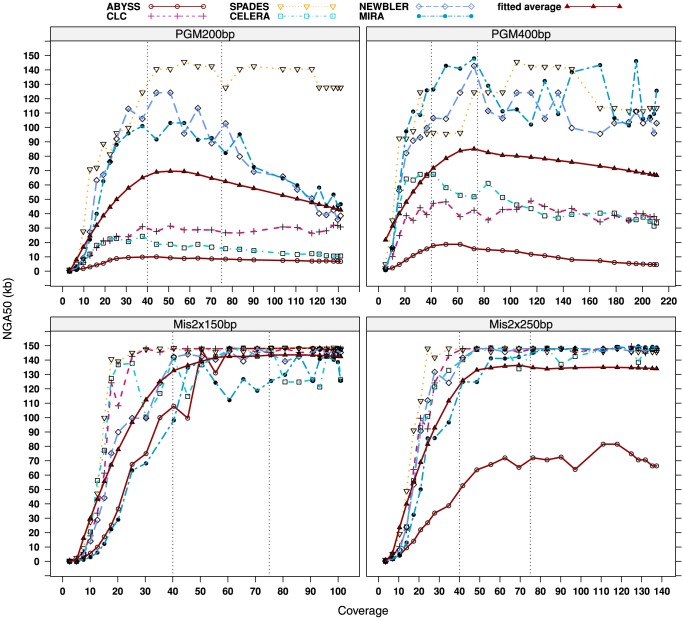
Effect of the depth of coverage on the assembly efficiency measured by NGA50 sizes based on randomly sub-sampled *E. coli* Sakai data sets. The coverage is referring to the average depth each genomic position is covered by the sequencing reads and not to the average depth of coverage the assemblies are actually reaching. The fitted average is, for each data set, the mean of all NGA50 lengths at each coverage fitted to a nonlinear local regression model. Sub-sampling was done in steps as a percentage of the original full sample size; hence, the x-axis ranges of the four sub-plots differ. The dotted vertical lines mark the finally used 40-fold (PGM 200 bp) and 75-fold coverage limits (PGM 400 bp, MiSeq 2×150 bp and MiSeq 2×250 bp).

Independent of the data set and the assembler, we found out that for each assembler and data set a window of reasonable coverage can be identified which is confined by a lower and upper bound, which mark the areas of inappropriate coverage (**Figure 1**). Given the *E. coli* Sakai data sets, too low coverages result in inferior NGA50 lengths progressively improving with increasing coverage, whereas above the upper bound a further increase in the depth of coverage does not necessarily effects superior assemblies. For the PGM platform oversampling, i.e. too high coverage, can even have a negative impact on the assembly especially if combined with OLC assemblers. Here, NGA50 lengths are constantly falling after reaching a specific maximum. This effect is apparent for both the PGM 200 bp and PGM 400 bp data, albeit the global coverage maxima are higher for the 400 bp (between 40- and 100-fold coverage) than for the 200 bp data sets (between 40- and 50-fold coverage). The MiSeq platform is not susceptible to oversampling. After reaching sufficient coverage, NGA50 lengths are mostly saturated and show only moderate improvement or degradation, marking the upper bound of appropriate coverage for the MiSeq data sets. These findings are in good accordance with the other data sets and with other metrics, as e.g. the number of mis-assemblies (local and non-local mis-assemblies) or the number of assembly errors (insertions, deletions and substitutions) and is further supported by another sub-sampling approach (see Supporting Information **Text S1** and **Figures S1–S5**). In order to achieve comparability between the data sets and the assemblers, we have selected two different cutoffs, reflecting the described upper coverage bounds, at which we sub-sample our data sets prior to any consecutive analysis. The 40-fold coverage limit was chosen for the PGM 200 bp data, whereas all MiSeq and the remaining PGM 400 bp data sets were sub-sampled at 75-fold depth of coverage. Even though these thresholds do not always represent individual global optima, they are not in favor or disfavor of any particular assembler or sequencing platform and in good accordance with previous findings [9], [15], [33].

### K-mer parametrization of de Bruijn Graph assemblers

Generally stated, DBG assemblers break raw sequencing reads into a set of k-mers and construct a graph by connecting the suffix and prefix nodes of overlapping k-mers by edges. By that, the size of the DBG depends only on the length of the k-mer and not on the initial read length distribution. Albeit this has a beneficial effect on the memory footprint and graph traversing time, it comes at the price for another parameter optimization step. Surprisingly, we are not aware of any study that tried to analyze this optimization problem extensively, nor were it a topic in previous assembly evaluation surveys, even though its existence is common knowledge in the assembly community. To meet this shortcoming, we have examined the effect of the k-mer parameter *k* on all data sets and all DBG assemblers for which a *k* needs to be specified (ABYSS, SOAP2 and VELVET) by iteratively running these assemblers at all supported values of *k*. Two main effects can be seen. First, the choice of an optimal *k* is depending on the underlying data set. Assemblages of different data sets using the same assembler provided different best performing values of *k*. Second, the optimal *k* also depends on the assembler and strongly varies even though the same data set was assembled. An approximation of an optimal *k* is further complicated by the fact that already the results based on one assembler and one data set show more than one local optima along the full spectrum of *k*. Hence, best performing k-mer parameters for the consecutive assemblies were determined by optimizing over the whole possible k-mer spectrum. For PGM data sets a k-mer optimization is less reasonable. Even though comparable pattern as for the MiSeq data were observed, overall unsatisfactory results were obtained independent of *k*. For an in depth description on the K-mer parametrization of de Bruijn Graph assemblers see the Supporting Information (**Text S1** and **Figures S6** and **S7**).

### Evaluation of *de novo* assemblies

**Figure 2:**
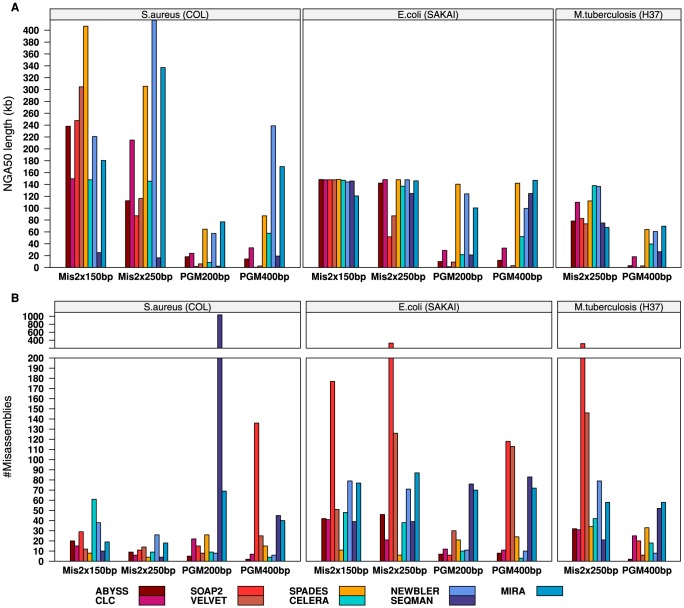
Comparison between the *de novo* genome assemblies based on the NGA50 length and the number of mis-assemblies. The NGA50 length (**A**, in kilobases) and the number of mis-assemblies (**B**, combining local and non-local mis-assemblies) on the y-axis are either contig or scaffold based, respectively. Scaffolds for MiSeq 2×150 bp and MiSeq 2×250 bp assemblies obtained by ABYSS, CELERA, CLC, NEWBLER, SOAP2, SPADES, and VELVET; contigs for MiSeq assemblies obtained by MIRA and SEQMAN as well as for all PGM assemblies. The second plot (**B**) is further divided into two plot rows where the upper row has an altered y-axis scale only showing high rates of mis-assemblies ranging from two hundred up to thousand.

**Figure 3:**
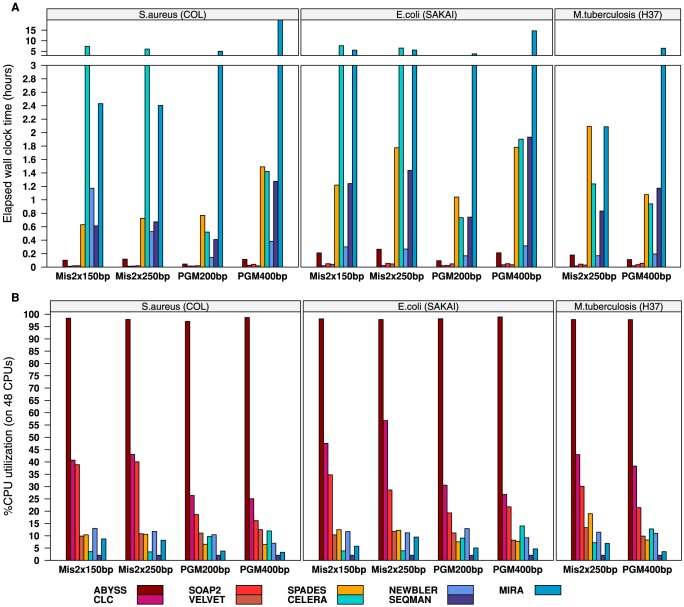
Computing time of *de novo* genome assemblies. Based on the elapsed wall clock time (**A**, in hours) and the total CPU utilization (**B**, in percent and relative to the 48 available CPU cores of the executing compute host). With regard to the CPU utilization, all assemblies have been instructed via proper parameterization to make maximal use of the 48 available CPU cores. The only exceptions to this were SEQMAN, which does not support parallelization, and CELERA, which due to configuration constraints has altering concurrency and multi-threading parameters for different internal processes. For DBG assemblers only run time and CPU utilization of the single assemblies with the best performing k-mer parameter are shown and not the summation of the full k-mer optimization procedure (for SPADES and CLC this is equivalent).

Combining ten data sets and nine assemblers we have generated 90 *de novo* assemblies. For every single assembly, the assembly effort was measured using QUAST [31]. Results were transformed into a spread-sheet readable tabulator separated file format and stored in addition to the complete assembly output, log files, and run time benchmarking results. In **Figure 2** we present the results of each assembly using the following two key metrics: the NGA50 length and the number of mis-assemblies. As mentioned before, these figures show either scaffolds or contigs according to the data, i.e. scaffolds for all MiSeq assemblies except for MIRA and SEQMAN and contigs for all others including the PGM assemblies. Similarly, **Figure 3** shows the central aspect of the running time benchmark, i.e. the wall clock time and the average system utilization. Details about specific assembly parameters and the full execution pipeline are given in the Supporting Information (**Text S1**) or can be looked up at the following project site: ftp://ftp.cebitec.uni-bielefeld.de/pub/GABenchToB.

At this repository, also the 90 assembly results together with all computed assembly metrics can be accessed. In addition to this core set, each of the more than two thousand assemblies used for the coverage and k-mer parameter optimization are deposited here, with the limitation that not for all of those assembly and run time metrics are available.

## Discussion

One of our main goals was to provide the research community with a practice-oriented *de novo* assembly evaluation of bacterial genomes sequenced by benchtop instruments. In this spirit, our study is intended to be more than a pure assembler comparison following the question which assembler performs best. Also, it is not a sequencing platform comparison trying to answer which platform allows for the best assemblies. Instead, given a diverse set of sequencing data, we identified those assemblers, which are best suited to handle individually different data sets and meet specific requirements such as the least amount of mis-assemblies or the fastest compute time.

Consequently, given the assembly results in **Figure 2** and **Figure 3**, none of the assemblers emerges as the overall winner. The individual assembler performance as given by the NGA50 length strongly depends on the nature of the data (**Figure 2A**). For MiSeq reads the assembler selection is less restricted than it is for PGM reads. Both, DBG and OLC assemblers are generally applicable on the MiSeq data sets. The *E. coli* MiSeq 2×150 bp and 2×250 bp assemblies, for example, yield NGA50 values of the same magnitude (about 155 kb) for both approaches. The highest MiSeq 2×150 bp *S. aureus* NGA50 lengths originate from DBG assemblers, whereas OLC assemblers performed better on the MiSeq 2×250 bp *S. aureus* and *M. tuberculosis* data. In contrast, assembled PGM data yielded better results more often when using OLC than DBG assemblers. Comparing the sum of all NGA50 lengths of the DBG assembled PGM 200 bp and 400 bp data sets with those using OLC, a more than 2-fold increase can be observed (719 megabases (Mb) compared to 1518 Mb). The only exception to this is the DBG SPADES assembler, which achieves NGA50 lengths on a par with the OLC assemblers. Omitting SPADES from this comparison, the proportion changes from a 2-fold increase to an over 6-fold increase (220 Mb compared to 1518 Mb), clearly showing the difficulties DBG assemblers have while dealing with PGM data. Considering the NGA50 length, we want to highlight the DBG assembler SPADES and the OLC assemblers NEWBLER and MIRA, as they repeatedly perform above average in their category and are more robust with respect to the kind of sequencing data.

Laying focus on the number of mis-assemblies, a different picture arises (**Figure 2B**). The NGA50 is the NG50 length after contigs have been split at each observed mis-assembly position. Resting on this relation, the naïve assumption would be that lots of mis-assemblies are directly reflected by a low NGA50 and vice versa. However, here a high and low mis-assembly rates could be observed independent of the NGA50 length for both assembler approaches across all data sets. The highest rate of mis-assemblies originates from an assembly that can be, measured against the NGA50, declared as failed (*S. aureus* PGM200 bp SEQMAN with NGA50 of 2292 bp). In contrast, some assemblies with also a very low NGA50 show very few mis-assemblies (e.g., all PGM ABYSS assemblies). However, discussing rates of mis-assemblies for assemblies with extremely low NGA50 values has little meaning. Assemblies with a reasonable NGA50 length again show both, i.e. a high (e.g., the SOAP2 and VELVET MiSeq *E. coli* and *M. tuberculosis* assemblies) and low (e.g., the SPADES MiSeq *E. coli* assemblies) rate of mis-assemblies. Of those, three assemblers show the most consistent pattern of mis-assemblies. While SPADES produced the least mis-assemblies in total across all data sets and sequencing platforms, NEWBLER produced high rates of mis-assemblies only when dealing with MiSeq data, and MIRA had one of the most miss-assembled contigs across all platforms. For all other assemblers, the number of mis-assemblies depended on the processed data and varies from very high to very low. Moreover, the NGA50 metric shows no significant correlation with the mis-assembly rates (data not shown). This implies, that a low NGA50 value is not necessarily equivalent to many mis-assemblies. Instead, it simply indicates a comparably inferior assembly, either due to many mis-assemblies or low assembly contiguity. These findings are also consistent with other metrics, e.g. the amount of fully covered genes and the number of assembly errors (insertions, deletions, and substitutions; **Figure S8**).

Genomes of extreme GC-rich or GC-poor bacteria are known to be challenging for genome assemblers as amplification biases of GC-poor or GC-rich regions can result in uneven genome coverage. Here, a weak relationship between the genomic GC-content and assembly contiguity could be found. Assemblages of the GC-poor *S. aureus* genome (33% GC) tend to reach higher NGA50 lengths than the *E. coli* (51% GC) and the GC-rich *M. tuberculosis* (66% GC) genome, for which on average the lowest NGA50 lengths were achieved. Likewise, the *S. aureus* assemblies exhibited fewer mis-assemblies than those of the other two genomes. However, the relatively inferior *M. tuberculosis* assemblies cannot be entirely explained by increased mis-assemblies, as they are comparable to the ones based on the *E. coli* genome. Instead, this discrepancy might be explained by the finding that extreme GC-rich regions are especially difficult to amplify [34], possibly lowering the assembly completeness and by that the contiguity. Despite these findings, a general conclusion on the effect of the GC-content on genome assemblies cannot be drawn, as this would require a broader range of differing genomes to be analyzed. In addition, the differences shown here are not clear enough and for all genomes a successful assembly could be generated. This is also supported by a previous study, which reports that the degree of a GC bias, the factor most influencing the assembly contiguity, correlates neither with the mean GC content nor with the standard deviation of GC content of a genome [35]. In the same study it could also be shown that a sufficient depth of coverage can compensate for a GC bias, which may explain the comparatively low differences observed here with regard to the sub-sampling optimized approach used in our study.

One neglected aspect in preceding assembler evaluation studies has been the computational cost of an assembly. By measuring the wall clock time of all assembly processes we observed a great discrepancy between DBG and OLC assemblers (**Figure 3A**). With the exception of SPADES, all DBG assemblers finished within less than 20 minutes, the majority even within less than five minutes, whereas OLC assemblers took from eight minutes up to 20 hours. NEWBLER is to be highlighted positively by showing consistently the shortest run time of the OLC assemblers. CELERA and MIRA, on the contrary, repeatedly exceeded run times of three hours. However, for all DBG assemblers only the run time of a single assembly effort is shown, i.e. of the single assembly call resulting in the highest N50 length (**Table S1**). So the total wall clock time for an entire assembly project needs to be adjusted with respect to the chosen k-mer optimization strategy and may markedly reduce the outstanding performance of the DBG assemblers. Exceptions to this are the assemblers CLC and SPADES as both do not rely on an external k-mer optimization step. Thus, their run times are equivalent to the overall needed run times. In this sense, CLC outperforms all other assemblers many times over. SPADES prolonged execution times, in contrast, can be explained by its operating procedures, as in the case of MiSeq PE data six and for PGM data five consecutive assemblies were performed internally.

A good indicator showing to which extent an assembler benefits from a parallelized computing environment is the average CPU utilization (**Figure 3B**). Surprisingly, only ABYSS is nearly full parallelized. All other assemblers have an average CPU utilization below 50%, which means that of the 48 available and requested CPUs, on average, only half of them were used during the full assembly procedure. Next to ABYSS, CLC and SOAP2 show the second and third best CPU utilizations, respectively. Also CLC and SOAP2 have a higher utilization if operating on MiSeq data than on PGM, showing that they are obviously optimized for Illumina data. All other assemblers have a total CPU utilization below 20%. This implies that the running time of those assemblers cannot be increased considerably by assigning more CPU cores. However, a low CPU utilization does not necessarily result in long running times, as demonstrated by VELVET, which always finished in less than four minutes but never exceeded a comparably low workload of 13.3%. One aspect negatively influencing the CPU utilization are input and output (I/O) operations, causing processes to enter a waiting state. Here, most critical are I/O operations caused by swapping when system memory is insufficient. The memory usage of our assemblies was very different but system memory was always sufficient and no assembly process was waiting because of swapping (**Figure S9**). However, some assemblers (e.g., MIRA, SPADES and CELERA) utilize or demand considerably more memory than others, which should be considered before an assembly attempt in order to circumvent swapping. One possible solution, besides the extension of the system memory, is to make use of specific memory constraining parameters that memory intensive assemblers usually offer. With regard to the average system utilization, we want to clarify that the assemblers are different in terms of the implemented parallelization and the internal assembly pipeline. Thus, a low CPU utilization does not mean that the entire assembly is inadequately parallelized, but that, for instance, parts of the assembly are constrained to system I/O (e.g., because of data pre- and post-processing), resulting in wait times of the depending processes. Therefore, this metric should not be considered as a fixed upper limit of the parallelization capabilities of each assembler. Instead, it reflects to what extent an assembler scales, in terms of run time, with the provided computing power, and helps to find a suitable combination between assemblers and the available hardware infrastructure.

One central aspect of this study was to compare the assemblies from a practical point of view. By that, we omitted additional data processing steps like, for instance, error correction methods. However, to respond to the question whether upstream error correction is reasonable and which assembler would profit from such methods we have exemplary used two different read correction methods on our data sets (BayesHammer from SPAdes [23] for MiSeq data and Coral version 1.4 [36] for PGM data) and compared the consecutive assemblies with those using uncorrected data (**Figure S10**). In essence, the observed differences are marginal. Even for those assemblers that do not include an error correcting pre-processing step (e.g., SOAP2, VELVET and NEWBLER) the beneficial effects are, albeit partially present, not altering their individual performance.

In this evaluation, we did not perform extensive parameter optimization, i.e. whenever possible we used default parameters. For mandatory or data specific parameters without default values we have chosen appropriate values (details given in **Text S1**). The only exception to this was the k-mer parameter optimization. We have shown that an optimal k-mer highly depends on both the assembler as well as the given data set. This implies that unless the assembler itself offers a suitable k-mer parameter estimation (as e.g., CLC) or default values (as e.g., SPADES), currently the best solution is to pursue a trial-and-error approach. Of course, this problem is not described for the first time and algorithms have been developed to predict optimal k-mer parameters *a priori*. KmerGenie, for instance, uses a heuristic to generate k-mer abundance histograms in order to estimate the best possible k-mer value [37]. However, for our data sets the optimal k-mer as predicted by KmerGenie did not matched the k-mer length at which the best assembly was achieved (data not shown). This may be caused by parameter estimations inferred only from the input data, which cannot take into account assembler peculiarities. Still, k-mer estimation may prove useful for other data sets and, especially due to quick heuristics, it is to be preferred over randomly chosen or alleged established k-mer lengths. Parameters proven successful in the past may not be adequate for new assembly problems. The downsides of the trial-and-error approach, in turn, are drastically increased running times countering the speed advantage of DBG assemblers. Besides the k-mer parameter, testing and comparing various parameter settings for each assembler is possible beyond the scope of every evaluation effort simply due to the high computational cost. Moreover, results of comparisons in which assemblies were highly optimized are hard to transfer to other application scenarios, weakening the overall conclusions of such a comparison. Therefore, good default parameters and standardized recipes are needed to support unbiased and useful comparisons. Every evaluation is affected by the applied evaluation procedure and the used metrics. Therefore, the results of this study cannot be interpreted as final principles to rule in or out individual assemblers, but to give a general advice which assemblers to shortlist in consideration of an upcoming bacterial genome assembly. Nevertheless, given the scope of our evaluation we want to highlight some promising combinations between assemblers and bacterial BS data. The most obvious recommendation is to pair different BS platforms with specific assembler approaches, i.e. PGM data is better combined with OLC assemblers whereas MiSeq data shows stronger preferences towards DBG assemblers. The only exception here is the CELERA assembler, which consistently performs better on MiSeq than on PGM data. For MiSeq data, promising DBG assemblers to begin with are SPADES and CLC. Both assemblers offer good performing default k-mer parameters, are generally easy to execute and show one of the highest NGA50 and lowest mis-assembly rates among the DBG assemblers. In addition, SPADES also performed best on PGM data among DBG assemblers. The CLC assembler shows the least memory footprint and the notably fastest running time of all assemblers, which qualifies it for quick and reasonable draft assemblies. Given MiSeq data and a highly parallelized computing infrastructure, the ABYSS assembler might also be an option, as it generates good assembly results while offering the best CPU utilization. For PGM data, researchers should consider to sub-sample their data to a coverage between 40 and 80-fold prior to an assembly in order to prevent negative oversampling effects. The two OLC assemblers that are a good entry point for PGM data sets are MIRA and NEWBLER. MIRA is able to achieve very high NGA50 values particular at higher coverages but comes at the price of more mis-assemblies and a longer running time. NEWBLER, in contrast, convinces with the shortest running time of all OLC assemblers and a low rate of mis-assemblies. Finally, it is to be noted that there is still a great discrepancy between researchers who are developing or evaluating genome assemblers and researchers who simply want to use them. The former ones take great care to avoid exaggerated generalizations, like to conclude that a particular assembler provides the best assemblies. The latter ones, on the other hand, are confronted with concrete application scenarios and therefore require decision-making support without needing to perform extensive evaluations personally. Therefore, evaluations should consider realistic assembly scenarios and include assembly metrics that summarize several assembly features into easy to communicate metrics.

## Methods

### Library preparation and sequencing

Growing and DNA extraction of the entero-haemorrhagic *Escherichia coli* (EHEC) O157:H7 Sakai Japanese 1996 outbreak strain was done as previously described [38]. For *Staphylococcus aureus* COL, an early methicillin-resistant strain originally isolated in a hospital in Colindale (United Kingdom) [39], growing conditions were the same as for *E. coli Sakai*. For effective cell lysis of *S. aureus* an additional step to the before mentioned DNA isolation protocol was essential. It was performed with Lysostaphin (Sigma-Aldrich, Taufkirchen, Germany, final concentration100 µg/ml), for 30 min at 37°C. Isolated DNA from *Mycobacterium tuberculosis H37Rv* was kindly provided by the group of Stefan Niemann (Forschungszentrum Borstel, Borstel, Germany). Growing and isolation of *M. tuberculosis* high molecular genomic DNA was performed as described earlier [40].

As described previously, sequencing of the *S. aureus* and *M. tuberculosis* strains was carried out for both the MiSeq 2×150 bp and 2×250 bp sequencing runs, respectively [2]. A minor modification was applied to the *M. tuberculosis* library, which was pooled after gel-extraction with other lane samples according to their molarity such that a 15 percent ratio was reached. Sequencing on the Ion Torrent PGM was also done as described before for the *E. coli* 200 bp and 400 bp sequencing libraries [2]. The two PGM *E. coli* 200 bp and 400 bp data sets were re-analyzed using the Torrent Sequencing Software (TSS) v3.0 and v3.4, respectively. PGM sequencing of *S. aureus* COL was performed in the same manner as it was described for *E. coli* Sakai with one minor modification, i.e. for the 400 bp sequencing run the TSS version was v3.2. Sequencing of *M. tuberculosis* was performed with the Ion PGM Template OT2 Kit (Life Technologies, Darmstadt, Germany) and the Ion PGM Sequencing 400 kit (Life Technologies) according to the manufacturer's instructions. Library preparation and quality controls were performed just as described for the *E. coli* Sakai 400 bp library. The TSS version was v3.6 for *M. tuberculosis* sequencing. Independent of the applied TSS version, the software parameters and quality filter criteria remained the same, i.e. the more stringent filter was enabled and the base recalibration was disabled.

### *De novo* genome assemblies

According to the findings on the genome coverage analysis, all data sets were sub-sampled prior to the *de novo* genome assembly. For the PGM 200 bp data a 40-fold coverage threshold was chosen, whereas PGM 400 bp and all MiSeq data were sub-sampled to 75-fold coverage. For each MiSeq data set insert size distribution was determined by mapping the PE reads to their corresponding reference using the *aln* and *sampe* module of BWA v0.5.10 [41]. Thereafter, the low and high boundaries of the insert sizes, as well as mean and standard deviation were calculated using the same method as implemented in BWA (**Table S2**). MiSeq FASTQ files were additionally edited to cope with special file format prerequisites of individual assemblers having different standards for the pairing information of PE reads.

All assemblies were independently computed on ten identical compute hosts, i.e. A+ Server 2024G-TRF (Super Micro Computer Inc., San Jones, CA, USA) equipped with four AMD Opteron 6168 processors, each with 1.90 GHz and 12 cores and in total 128 GB system memory. These machines were chosen as they provide reasonable computing power and a high degree of parallelization for asset costs below 10,000 Euros. Custom shell scripts were used to submit assembly bulk jobs to a Sun Grid Engine instance and the scheduler was utilized to consecutively distribute individual jobs among the ten compute hosts. Custom wrapper scripts for each assembler were used to prepare and initiate the actual assembly. To prevent network file system influences on the benchmarking results all necessary input data was copied to local storage prior to the assemblies. To guarantee that per compute host only one individual assembly job was executed at a time, each job was configured in such a way that it consumed the entire system resources regardless of the actual workload.

For recording run time statistics, each plain assembly process was wrapped once again using the ‘*/usr/bin/time -v* ’ system call. Additionally, at an interval of five seconds a ‘*ps -o ’‘%cpu =  %mem =  cputime =  etime =  nlwp =  size =  vsize =  sz =  rss =  lstart =  psr =  comm = *’ system call was invoked to monitor detailed processor and memory utilization of all assembly threads while the programs were executed. Details about used assembler versions are given in **Table 2**. After the assemblies were finished, QUAST v2.1 [31] was executed on the resulting contig or scaffold FASTA files with the minimum contig length filter ‘*–min-contig*’ set to 200 and the parameters ‘*–gage –genes –gene-finding* ’ enabled. QUAST utilizes MUMmer [42] to align contigs to a given reference and infer various assembly metrics. Regarding the measurement of the assembly accuracy, our evaluation effort follows in essence the GAGE-B evaluation [15]. In the following, the metrics used for our main comparison are explained briefly:

- NGA50 describes the length of the last contig that is taken from all assembled contigs sorted in descending order by contig length in such a way that the summarized length of this and all previously selected contigs have at least 50% of the size of the corresponding genome. Contigs were split at all mis-assembly positions prior to this calculation.
- The number of mis-assemblies, i.e. the combined number of relocations, translocations and inversions independent of the affected genomic area.
- Wall clock time refers to the time a plain assembly process takes in total from the very beginning to the very end. This measurement does not include any pre- or post-processing procedure and is not corrected for the system load, degree of parallelization or any software dependent idle time.
- The average system utilization *U* describes the degree of parallelization of an assembly process in percent and is defined as follows:
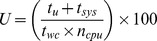

Where *t_u_* refers to the accumulated user time for which all CPUs were busy executing the assembly, *t_sys_* to the amount of time the CPUs spent on system calls on behalf of the assembly process, *t_wc_* to the wall clock time of the entire assembly and *n_cpu_* to the number of available CPUs.

An average system load of 100% corresponds to 100% work load of all 48 available CPU cores for the entire execution, illustrating maximum possible parallelization of all parts of an assembly. In contrast, a system load of 2.08% means that for the entire execution only one of the 48 available cores was occupied, which is synonymous to no parallelization. Finally, assembly and run time metrics were collected and processed using custom Perl and shell scripts. Plots were generated using the statistical software suite R v 2.9.10 [43] and the lattice R-package [44]. For an in-detail description of the complete bioinformatic analysis it is referred to the Supporting Information (**Text S1**).

## Supporting Information

Figure S1 **Effect of the depth of coverage on NGA50 lengths using random sub-sampling.** Shown are in rows the results of randomly sub-sampled *S. aureus*, *E. coli*, and *M. tuberculosis* data sets, respectively. The coverage is referring to the average depth each genomic position is covered by the sequencing reads and not on the average depth of coverage the assemblies are actually reaching. The dotted vertical lines mark the finally used 40-fold (PGM 200 bp) and 75-fold coverage limits (PGM 400 bp, MiSeq 2×150 bp and MiSeq 2×250 bp).
(PDF)

Figure S2 **Effect of the depth of coverage on mis-assemblies using random sub-sampling.** Shown are in rows the results of randomly sub-sampled *S. aureus*, *E. coli*, and *M. tuberculosis* data sets, respectively. Mis-assembly combines local and non-local mis-assemblies. The coverage is referring to the average depth each genomic position is covered by the sequencing reads and not on the average depth of coverage the assemblies are actually reaching. The dotted vertical lines mark the finally used 40-fold (PGM 200 bp) and 75-fold coverage limits (PGM 400 bp, MiSeq 2×150 bp and MiSeq 2×250 bp).
(PDF)

Figure S3 **Effect of the depth of coverage on assembly errors using random sub-sampling.** Shown are in rows the results of randomly sub-sampled *S. aureus*, *E. coli*, and *M. tuberculosis* data sets, respectively. Assembly error is summarizing substitutions, insertions, and deletions errors. The coverage is referring to the average depth each genomic position is covered by the sequencing reads and not on the average depth of coverage the assemblies are actually reaching. The dotted vertical lines mark the finally used 40-fold (PGM 200 bp) and 75-fold coverage limits (PGM 400 bp, MiSeq 2×150 bp and MiSeq 2×250 bp).
(PDF)

Figure S4 **Effect of the depth of coverage on NGA50 lengths using progressive sub-sampling.** Shown are in rows the results of progressively sub-sampled *E. coli* and *S. aureus* data sets, respectively. The coverage is referring to the average depth each genomic position is covered by the sequencing reads and not on the average depth of coverage the assemblies are actually reaching. The dotted vertical lines mark the finally used 40-fold (PGM 200 bp) and 75-fold coverage limits (PGM 400 bp, MiSeq 2×150 bp and MiSeq 2×250 bp).
(PDF)

Figure S5 **Effect of the depth of coverage on mis-assemblies using progressive sub-sampling.** Shown are in rows the results of progressively sub-sampled *E. coli* and *S. aureus* data sets, respectively. Mis-assembly combines local and non-local mis-assemblies. The coverage is referring to the average depth each genomic position is covered by the sequencing reads and not on the average depth of coverage the assemblies are actually reaching. The dotted vertical lines mark the finally used 40-fold (PGM 200 bp) and 75-fold coverage limits (PGM 400 bp, MiSeq 2×150 bp and MiSeq 2×250 bp).
(PDF)

Figure S6 **Effect of the k-mer size parameter on the NGA50 length.** Shown are values for the three de Bruijn Graph assembler ABYSS (**A**, **D**), SOAP2 (**B**, **E**), and VELVET (**C**, **F**). On the left side (**A**, **B**, **C**) using MiSeq 2×150 bp (dotted lines) and MiSeq 2×250 bp (solid lines); on the right side (**D**, **E**, **F**) using PGM 200 bp (dotted lines) and PGM 400 bp (solid lines) data sets of the *E. coli* (red), *M. tuberculosis* (blue), and *S. aureus* (green) genomes, respectively. For each line, the highest reached NGA50 length is indicated by a vertical arrow and the corresponding x- and y-values are given in the upper left legend.
(PDF)

Figure S7 **Effect of the k-mer size parameter on the NGA50 length of the SPADES assembler.** The assemblies were generated in two ways: using an increasing set of k-mer parameters where for each assembly process the NGA50 length of the last and final k-mer cycle is drawn (**A**); using the default set of k-mer parameters where the NGA50 length of all intermediate and the final k-mer cycle is drawn (**B**). MiSeq 2×150 bp (dark-red), MiSeq 2×250 bp (red), PGM 200 bp (green), and PGM 400 bp (dark-green) data sets of the *E. coli* (solid lines), *M. tuberculosis* (dot-dashed lines), and *S. aureus* (dashed lines) genomes are used, respectively. For each line, the highest reached NGA50 length is indicated by a vertical arrow and the corresponding x- and y-values are given in the upper left legend.
(PDF)

Figure S8 **Gene coverage and assembly error rates of** ***de novo*** **genome assemblies.** Based on the percentage of full covered genes (**A**) and the number of assembly errors (**B**, combining substitutions, insertions, and deletions). Full covered genes are completely covered positions in the reference genome where a gene annotation was provided (based on all chromosomal and plasmid genes). The numbers of assembly errors are either contig or scaffold based, respectively. Scaffolds for MiSeq 2×150 bp and MiSeq 2×250 bp assemblies obtained by ABYSS, CELERA, CLC, NEWBLER, SOAP2, SPADES, and VELVET; contigs for MiSeq assemblies obtained by MIRA and SEQMAN as well as for all PGM assemblies.
(PDF)

Figure S9 **Memory usage of** ***de novo*** **genome assemblies.** Shown is the maximum non-swapped physical memory, i.e. the peak resident size that an assembly process has used over the entire time. For assemblies running several processes or threads in parallel this value is calculated from the maximum summation of all concurrent processes at a specific time point. For the DBG assemblers ABYSS, SOAP2, and VELVET only the peak resident size of the best resulting k-mer parameter are shown and not the summation of all assemblies using different k-mer parameters.
(PDF)

Figure S10 **Effect of upstream error correction on** ***de novo*** **genome assemblies.** Compared are NGA50 lengths (in kilobase pairs) of assemblies without an upstream read based error correction (left side) with those based on error corrected reads (using BayesHammer on MiSeq data and Coral on PGM data; right side). The NGA50 length is either contig or scaffold based, respectively. Scaffolds for MiSeq 2×150 bp and MiSeq 2×250 bp assemblies obtained by ABYSS, CELERA, CLC, NEWBLER, SOAP2, SPADES, and VELVET; contigs for MiSeq assemblies obtained by MIRA and all PGM assemblies.
(PDF)

Table S1 **Determined optimal k-mer sizes for DBG assemblies with mandatory k-mer parameterization.**
(DOC)

Table S2 **Calculated insert-sizes of MiSeq sequencing libraries.**
(DOC)

Text S1 **Supporting Information to GABenchToB: A Genome Assembly Benchmark Tuned on Bacteria and Benchtop Sequencers.**
(PDF)
