# Correction: GABenchToB: A Genome Assembly Benchmark Tuned on Bacteria and Benchtop Sequencers

**DOI:** 10.1371/journal.pone.0118741

**Published:** 2015-03-19

**Authors:** The PLOS ONE Staff

Fig. 2 and Supporting S8 Figure are incorrect. Please view the corrected figures here.

**Fig 2:**
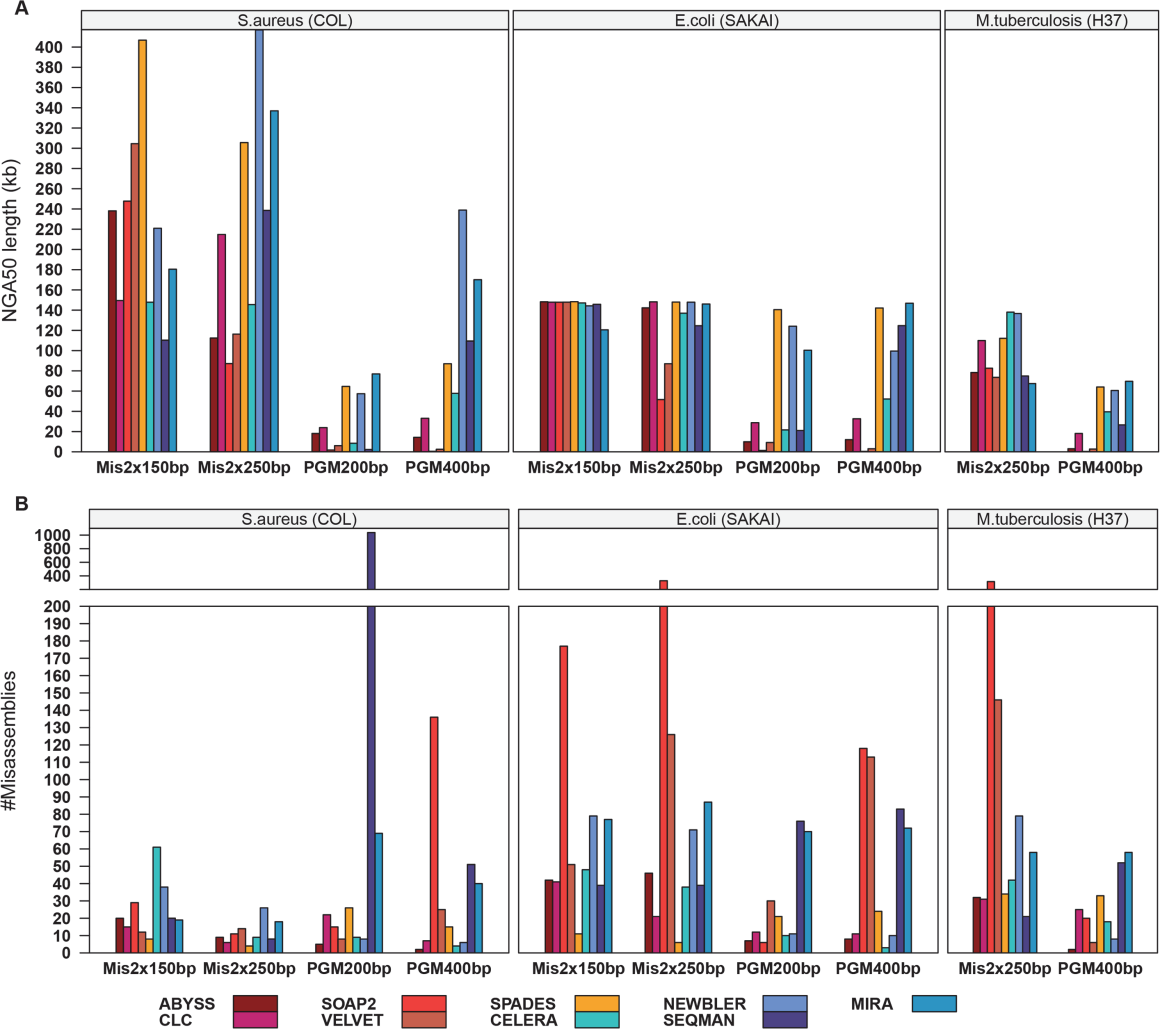
Comparison between the *de novo* genome assemblies based on the NGA50 length and the number of mis-assemblies. The NGA50 length (A, in kilobases) and the number of mis-assemblies (B, combining local and non-local mis-assemblies) on the y-axis are either contig or scaffold based, respectively. Scaffolds for MiSeq 2×150 bp and MiSeq 2×250 bp assemblies obtained by ABYSS, CELERA, CLC, NEWBLER, SOAP2, SPADES, and VELVET; contigs for MiSeq assemblies obtained by MIRA and SEQMAN as well as for all PGM assemblies. The second plot (B) is further divided into two plot rows where the upper row has an altered y-axis scale only showing high rates of mis-assemblies ranging from two hundred up to thousand.

## Supporting Information

S8 Figure Gene coverage and assembly error rates of *de novo* genome assemblies. Based on the percentage of full covered genes (A) and the number of assembly errors (B, combining substitutions, insertions, and deletions). Full covered genes are completely covered positions in the reference genome where a gene annotation was provided (based on all chromosomal and plasmid genes). The numbers of assembly errors are either contig or scaffold based, respectively. Scaffolds for MiSeq 2×150 bp and MiSeq 2×250 bp assemblies obtained by ABYSS, CELERA, CLC, NEWBLER, SOAP2, SPADES, and VELVET; contigs for MiSeq assemblies obtained by MIRA and SEQMAN as well as for all PGM assemblies. doi:10.1371/journal.pone.0107014.s008
(PDF)

## References

[1] Jünemann S, Prior K, Albersmeier A, Albaum S, Kalinowski J, Goesmann A, et al (2014) GABenchToB: A Genome Assembly Benchmark Tuned on Bacteria and Benchtop Sequencers. PLoS ONE 9(9): e107014 doi:10.1371/journal.pone.0107014 25198770 10.1371/journal.pone.0107014 PMC4157817

