# Correction: GABenchToB: A Genome Assembly Benchmark Tuned on Bacteria and Benchtop Sequencers

**DOI:** 10.1371/journal.pone.0299269

**Published:** 2024-02-15

**Authors:** Sebastian Jünemann, Karola Prior, Andreas Albersmeier, Stefan Albaum, Jörn Kalinowski, Alexander Goesmann, Jens Stoye, Dag Harmsen

In the Data Availability statement of this article [1, 2], the data repository link is no longer accessible. The correct data link is: https://gitlab.ub.uni-bielefeld.de/sjuenemann/gabenchtob. The updated Data Availability statement should therefore read: “The authors confirm that all data underlying the findings are fully available without restriction. Sequencing reads were deposited at the European Nucleotide Archive ENA and are available under the project accession number ERP006674. All underlying data are unrestricted and anonymously available at https://gitlab.ub.uni-bielefeld.de/sjuenemann/gabenchtob. In addition, all information required to reproduce the results, e.g. the full execution pipeline including all custom scripts, assembler installation configurations and run parameters, are also available through this link. Original raw sequencing data are available through ENA using the project accession number above.”

This new repository holds the study results as well as all the underlying data to replicate these, as was the case for the original FTP repository. However, due to disk quota limitations, intermediate results and the original raw sequencing data were removed from the repository. Both remain available at ENA/SRA as given by the accession numbers (ERP006674) in the article.
