# Supplementary figures and images for: GABenchToB: A Genome Assembly Benchmark Tuned on Bacteria and Benchtop Sequencers
Source: PLoS One. 2014 Sep 8;9(9):e107014. doi: 10.1371/journal.pone.0107014 (PMC4157817; doi:10.1371/journal.pone.0107014)

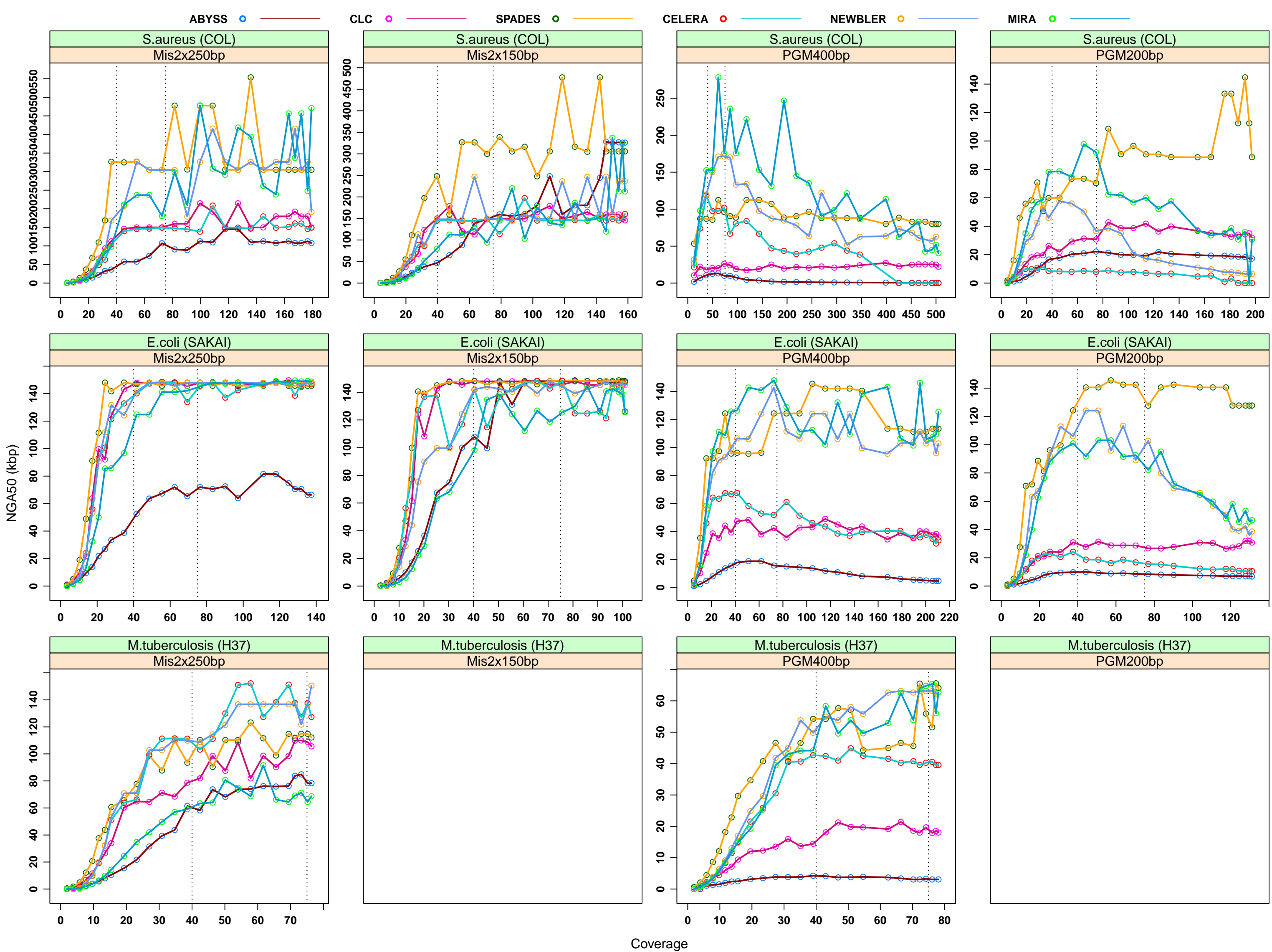

Supplement: Figure S1 — Effect of the depth of coverage on NGA50 lengths using random sub-sampling. Shown are in rows the results of randomly sub-sampled S. aureus, E. coli, and M. tuberculosis data sets, respectively. The coverage is referring to the average depth each genomic position is covered by the sequencing reads and not on the average depth of coverage the assemblies are actually reaching. The dotted vertical lines mark the finally used 40-fold (PGM 200 bp) and 75-fold coverage limits (PGM 400 bp, MiSeq 2×150 bp and MiSeq 2×250 bp). (PDF) [file pone.0107014.s001.pdf]

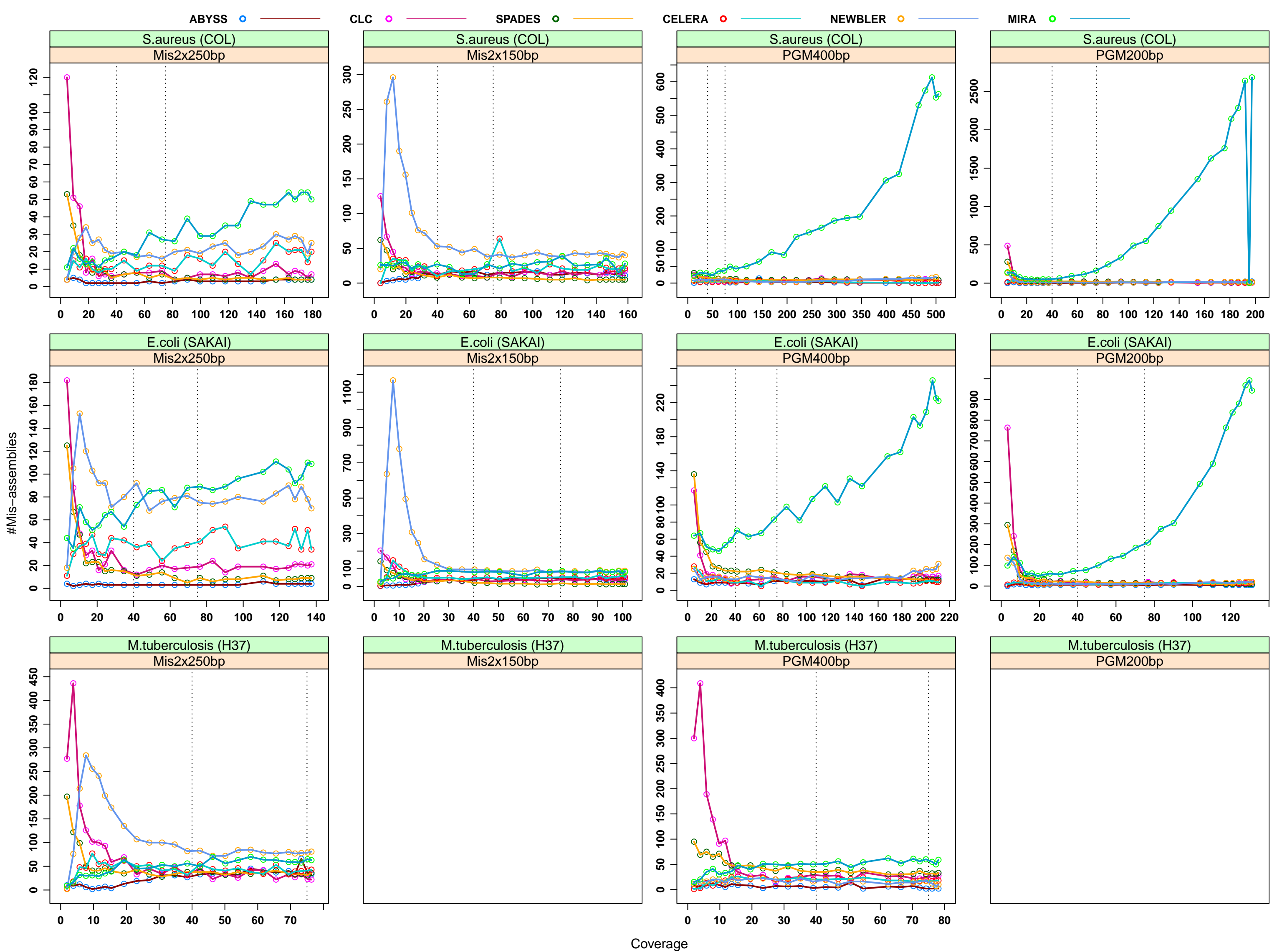

Supplement: Figure S2 — Effect of the depth of coverage on mis-assemblies using random sub-sampling. Shown are in rows the results of randomly sub-sampled S. aureus, E. coli, and M. tuberculosis data sets, respectively. Mis-assembly combines local and non-local mis-assemblies. The coverage is referring to the average depth each genomic position is covered by the sequencing reads and not on the average depth of coverage the assemblies are actually reaching. The dotted vertical lines mark the finally used 40-fold (PGM 200 bp) and 75-fold coverage limits (PGM 400 bp, MiSeq 2×150 bp and MiSeq 2×250 bp). (PDF) [file pone.0107014.s002.pdf]

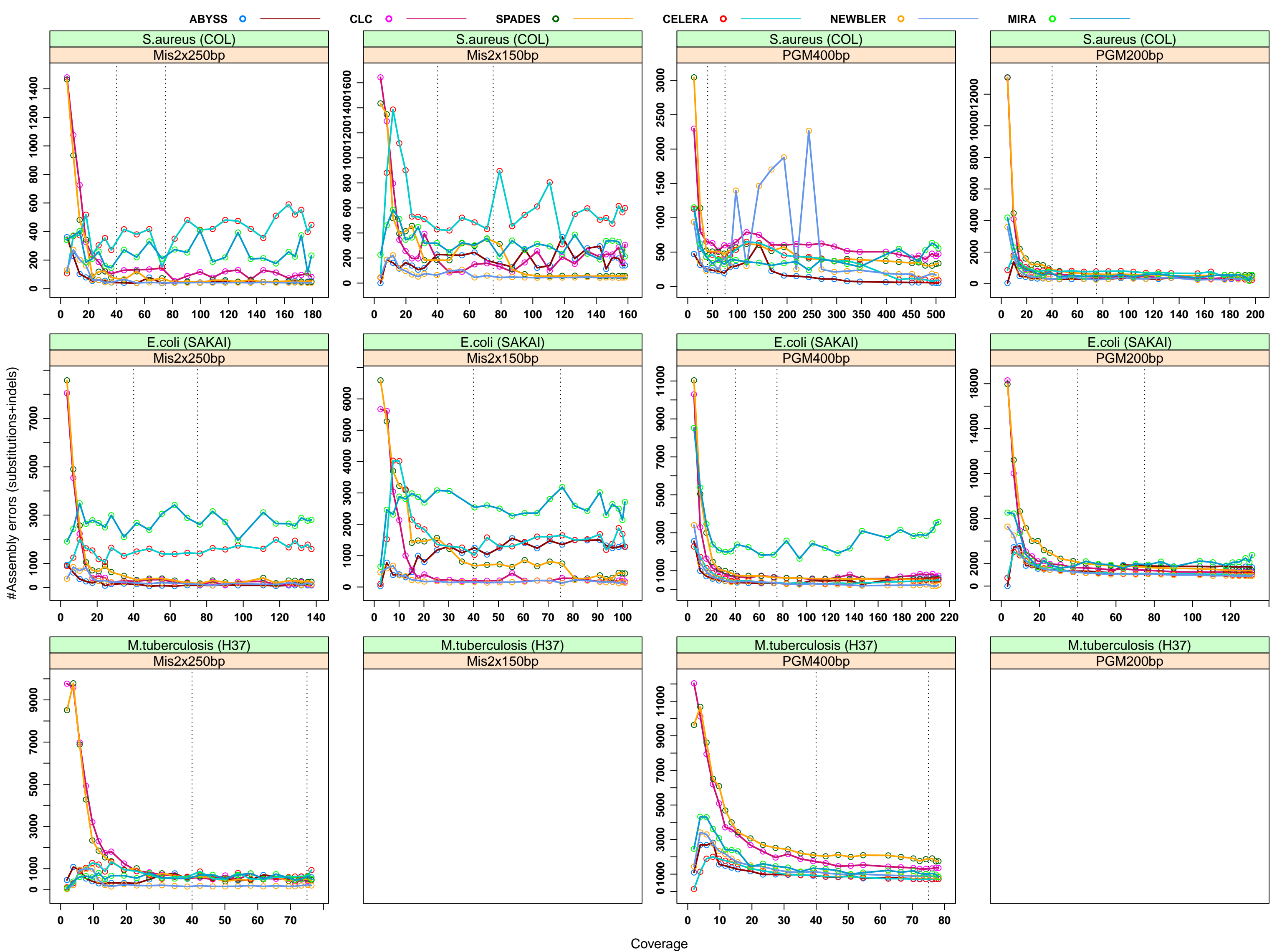

Supplement: Figure S3 — Effect of the depth of coverage on assembly errors using random sub-sampling. Shown are in rows the results of randomly sub-sampled S. aureus, E. coli, and M. tuberculosis data sets, respectively. Assembly error is summarizing substitutions, insertions, and deletions errors. The coverage is referring to the average depth each genomic position is covered by the sequencing reads and not on the average depth of coverage the assemblies are actually reaching. The dotted vertical lines mark the finally used 40-fold (PGM 200 bp) and 75-fold coverage limits (PGM 400 bp, MiSeq 2×150 bp and MiSeq 2×250 bp). (PDF) [file pone.0107014.s003.pdf]

ABYSS ○ — CLC ○ — SPADES ○ — CELERA ○ — NEWBLER ○ — MIRA ○

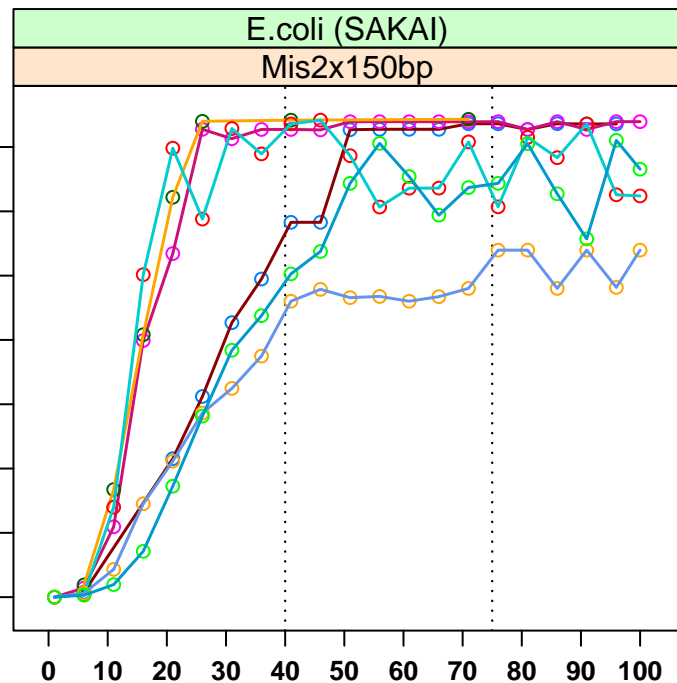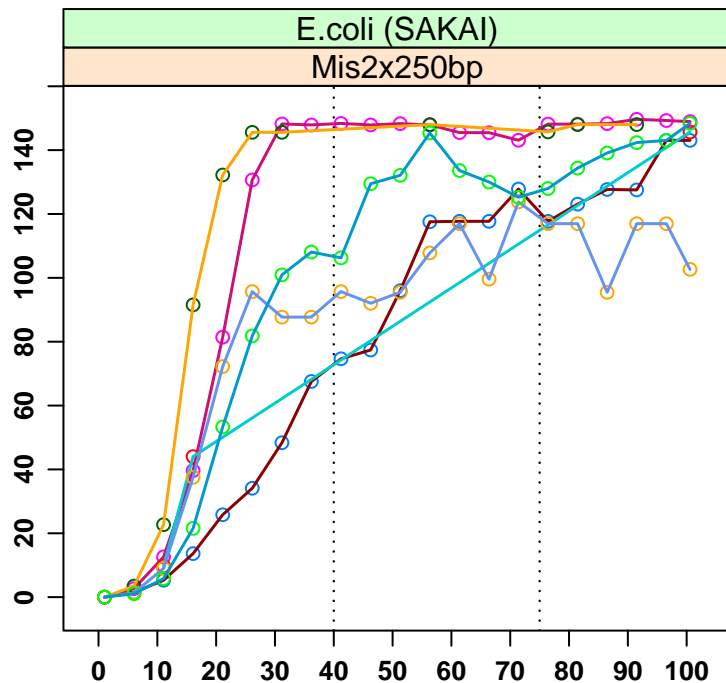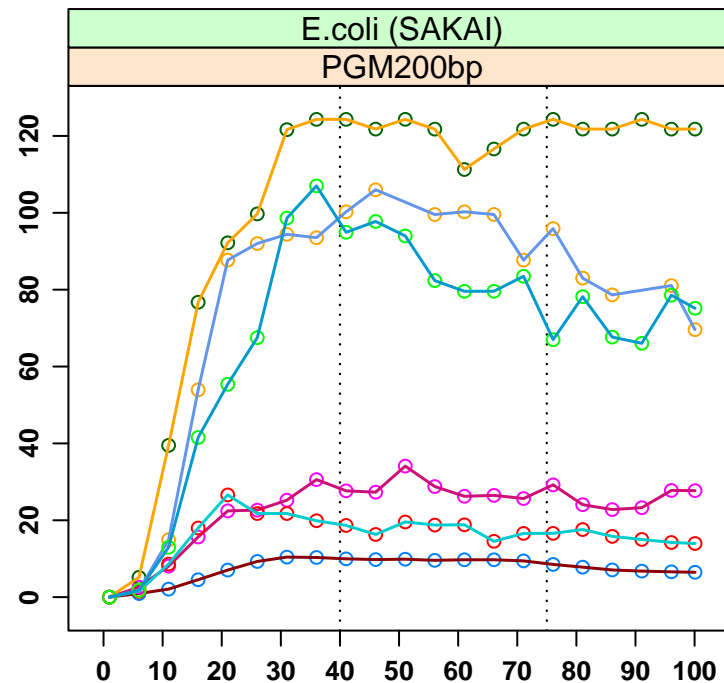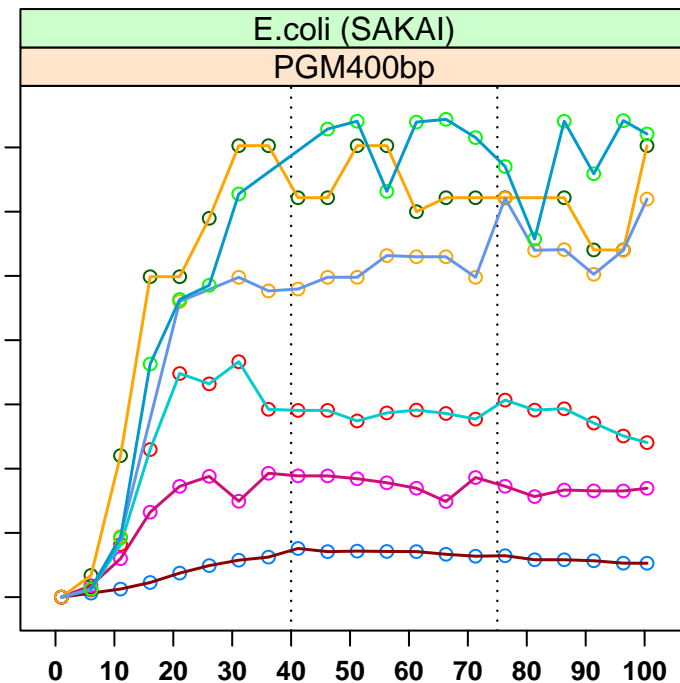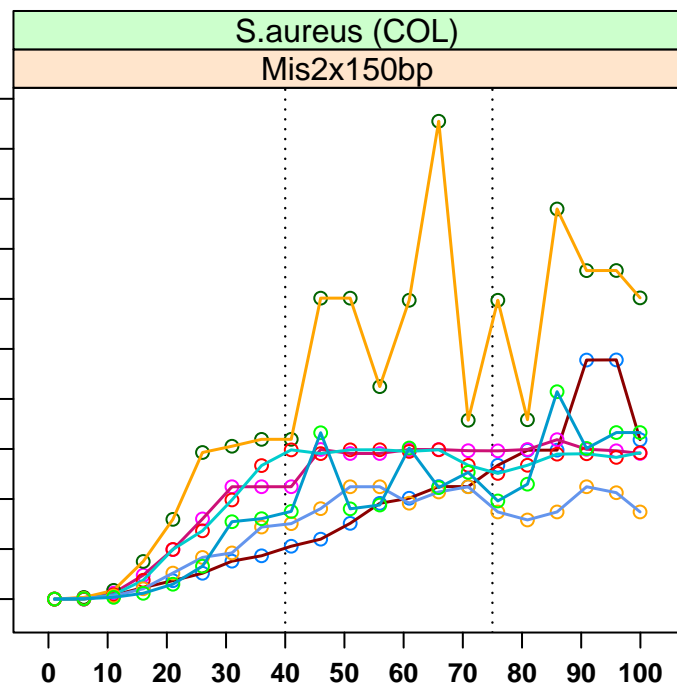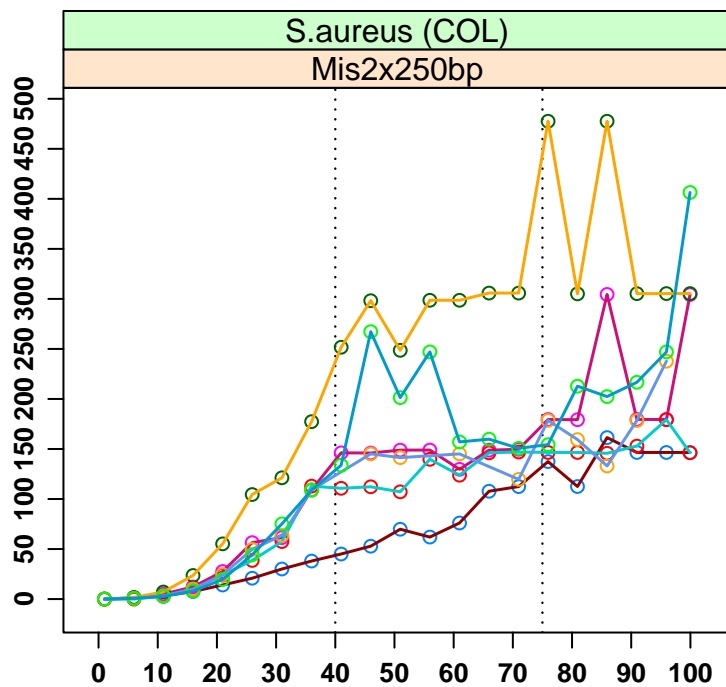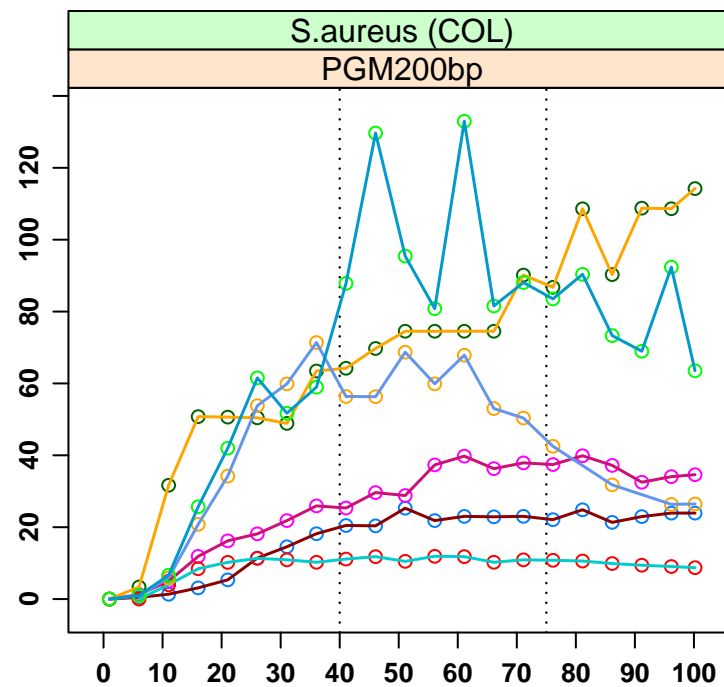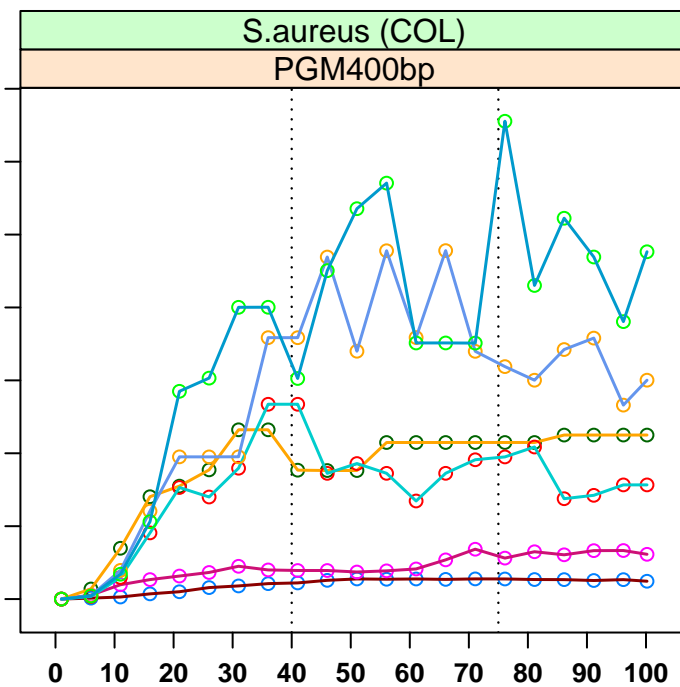

Supplement: Figure S4 — Effect of the depth of coverage on NGA50 lengths using progressive sub-sampling. Shown are in rows the results of progressively sub-sampled E. coli and S. aureus data sets, respectively. The coverage is referring to the average depth each genomic position is covered by the sequencing reads and not on the average depth of coverage the assemblies are actually reaching. The dotted vertical lines mark the finally used 40-fold (PGM 200 bp) and 75-fold coverage limits (PGM 400 bp, MiSeq 2×150 bp and MiSeq 2×250 bp). (PDF) [file pone.0107014.s004.pdf]

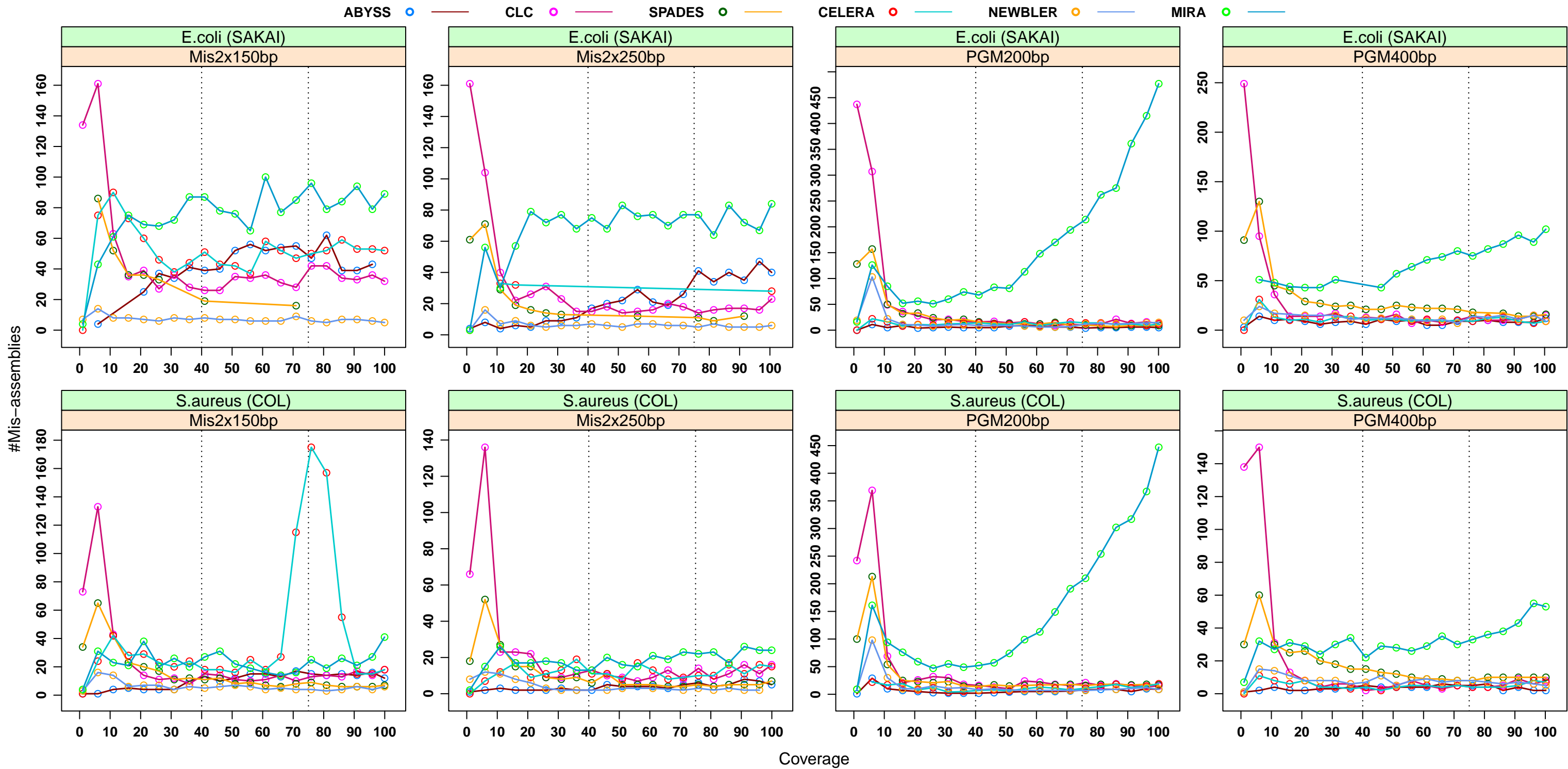

Supplement: Figure S5 — Effect of the depth of coverage on mis-assemblies using progressive sub-sampling. Shown are in rows the results of progressively sub-sampled E. coli and S. aureus data sets, respectively. Mis-assembly combines local and non-local mis-assemblies. The coverage is referring to the average depth each genomic position is covered by the sequencing reads and not on the average depth of coverage the assemblies are actually reaching. The dotted vertical lines mark the finally used 40-fold (PGM 200 bp) and 75-fold coverage limits (PGM 400 bp, MiSeq 2×150 bp and MiSeq 2×250 bp). (PDF) [file pone.0107014.s005.pdf]

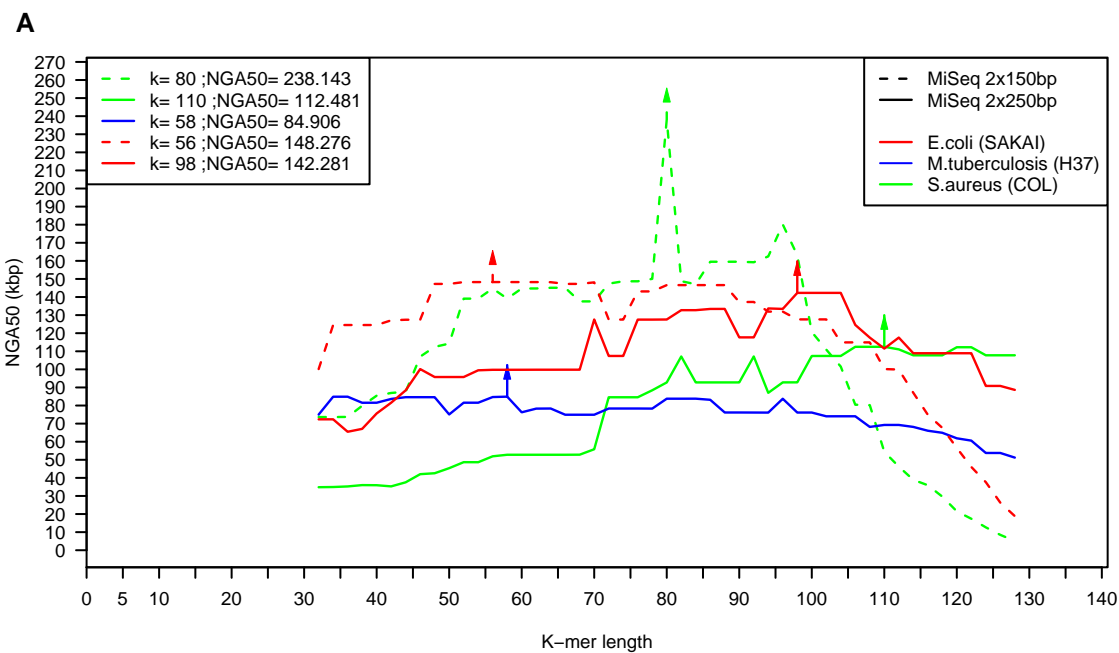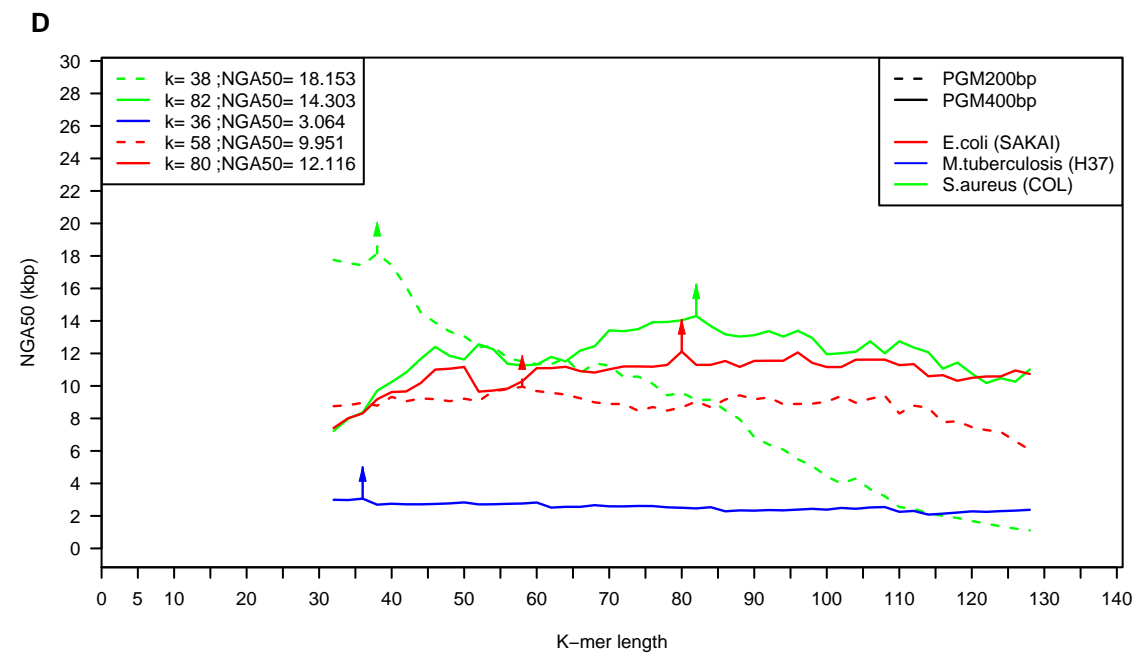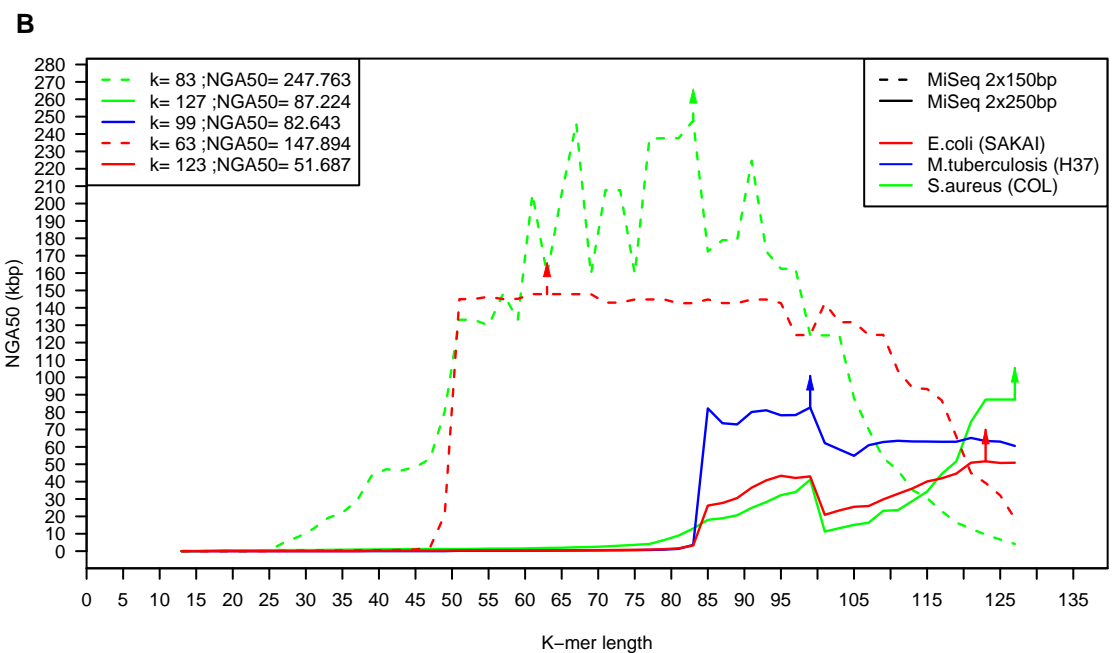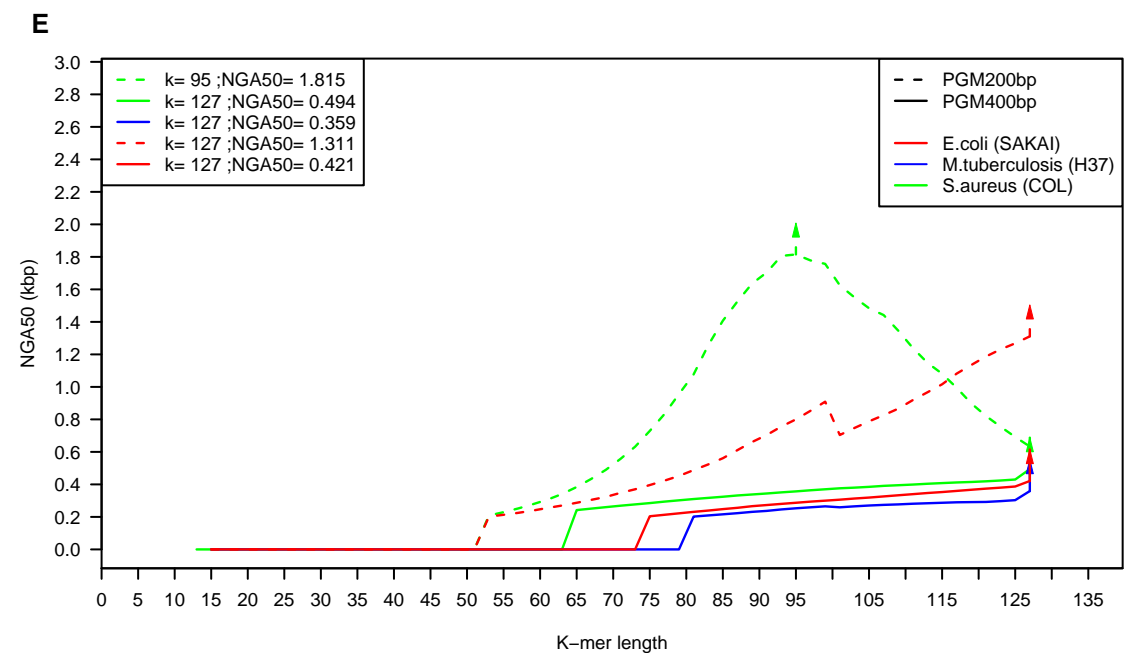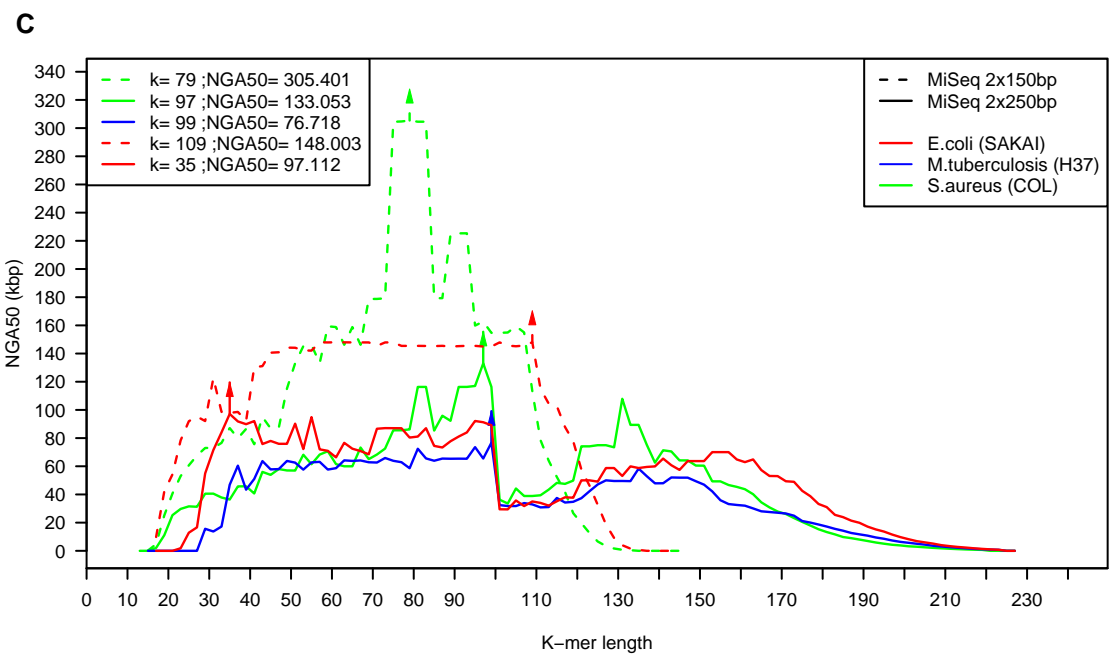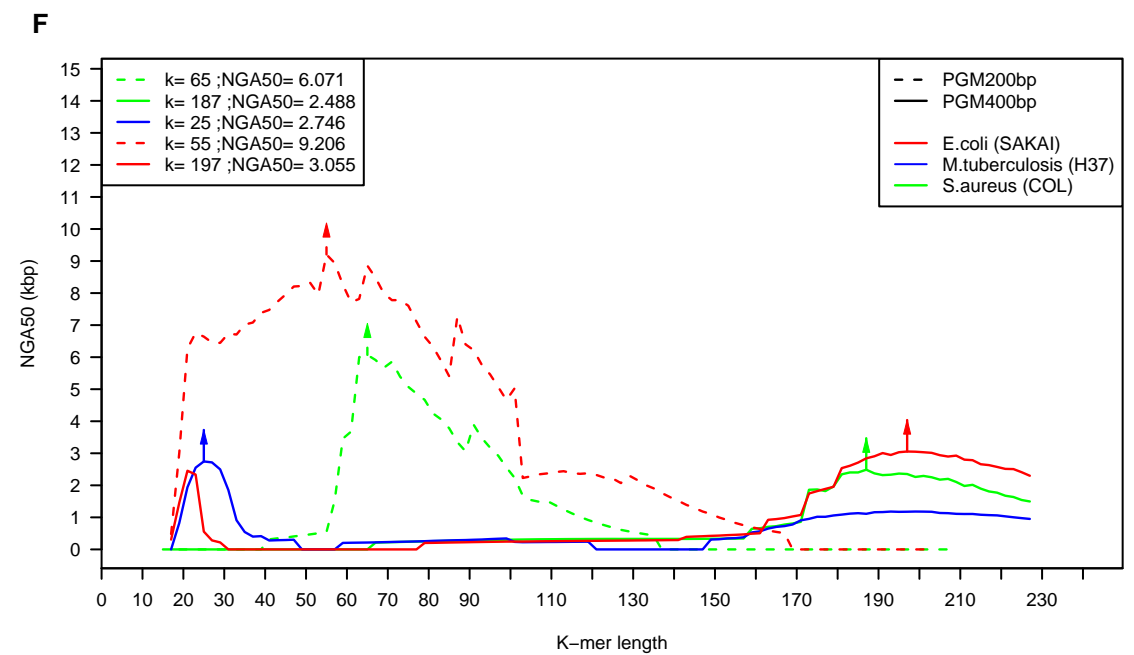

Supplement: Figure S6 — Effect of the k-mer size parameter on the NGA50 length. Shown are values for the three de Bruijn Graph assembler ABYSS (A, D), SOAP2 (B, E), and VELVET (C, F). On the left side (A, B, C) using MiSeq 2×150 bp (dotted lines) and MiSeq 2×250 bp (solid lines); on the right side (D, E, F) using PGM 200 bp (dotted lines) and PGM 400 bp (solid lines) data sets of the E. coli (red), M. tuberculosis (blue), and S. aureus (green) genomes, respectively. For each line, the highest reached NGA50 length is indicated by a vertical arrow and the corresponding x- and y-values are given in the upper left legend. (PDF) [file pone.0107014.s006.pdf]

**A**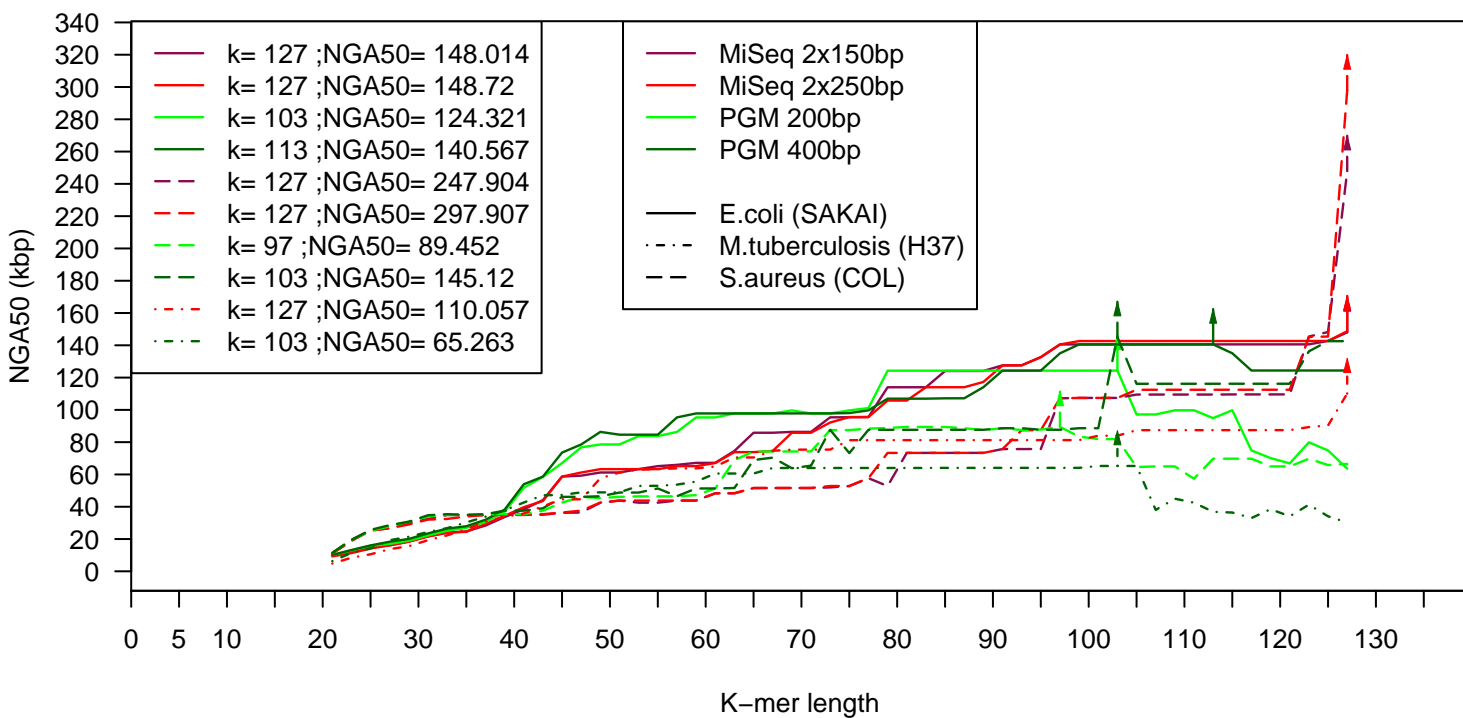**B**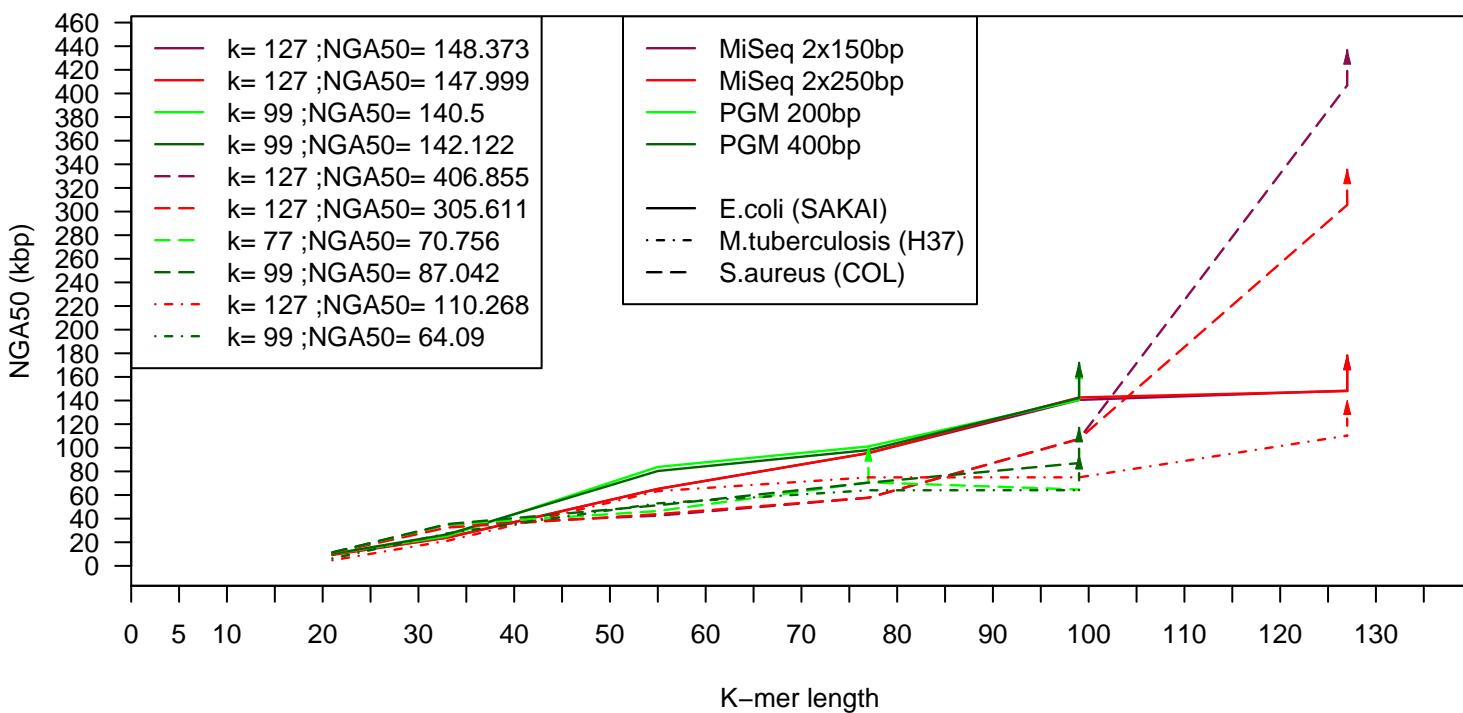

Supplement: Figure S7 — Effect of the k-mer size parameter on the NGA50 length of the SPADES assembler. The assemblies were generated in two ways: using an increasing set of k-mer parameters where for each assembly process the NGA50 length of the last and final k-mer cycle is drawn (A); using the default set of k-mer parameters where the NGA50 length of all intermediate and the final k-mer cycle is drawn (B). MiSeq 2×150 bp (dark-red), MiSeq 2×250 bp (red), PGM 200 bp (green), and PGM 400 bp (dark-green) data sets of the E. coli (solid lines), M. tuberculosis (dot-dashed lines), and S. aureus (dashed lines) genomes are used, respectively. For each line, the highest reached NGA50 length is indicated by a vertical arrow and the corresponding x- and y-values are given in the upper left legend. (PDF) [file pone.0107014.s007.pdf]

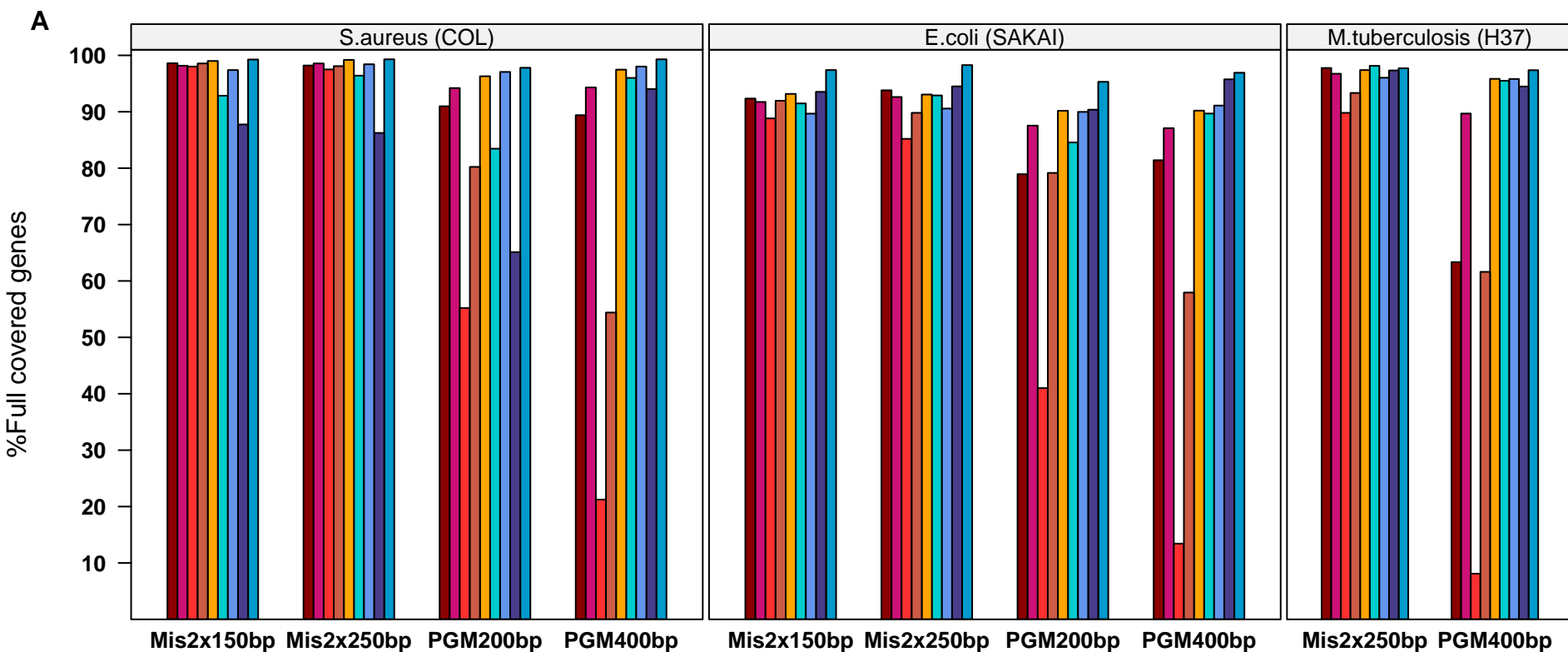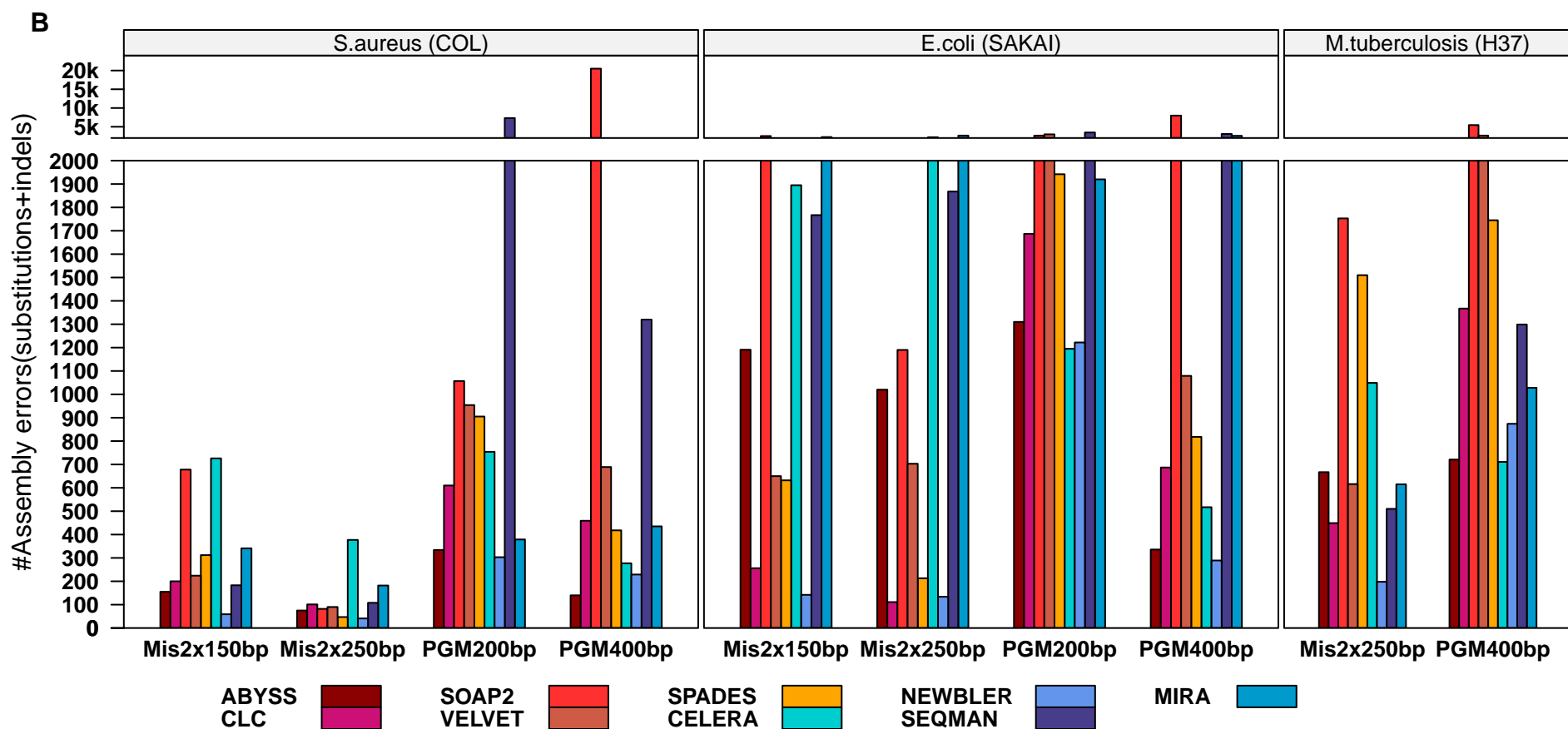

Supplement: Figure S8 — Gene coverage and assembly error rates of de novo genome assemblies. Based on the percentage of full covered genes (A) and the number of assembly errors (B, combining substitutions, insertions, and deletions). Full covered genes are completely covered positions in the reference genome where a gene annotation was provided (based on all chromosomal and plasmid genes). The numbers of assembly errors are either contig or scaffold based, respectively. Scaffolds for MiSeq 2×150 bp and MiSeq 2×250 bp assemblies obtained by ABYSS, CELERA, CLC, NEWBLER, SOAP2, SPADES, and VELVET; contigs for MiSeq assemblies obtained by MIRA and SEQMAN as well as for all PGM assemblies. (PDF) [file pone.0107014.s008.pdf]

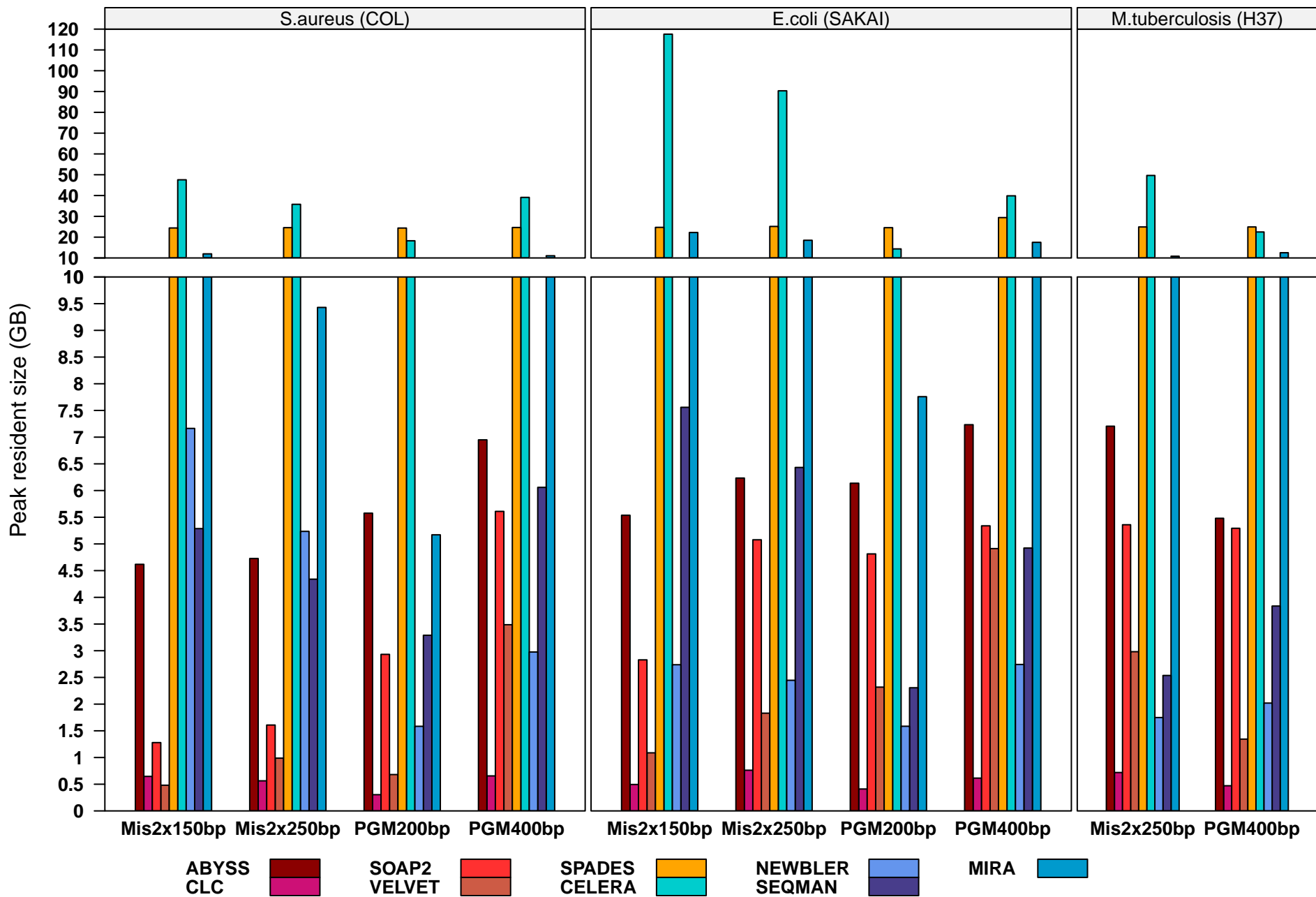

Supplement: Figure S9 — Memory usage of de novo genome assemblies. Shown is the maximum non-swapped physical memory, i.e. the peak resident size that an assembly process has used over the entire time. For assemblies running several processes or threads in parallel this value is calculated from the maximum summation of all concurrent processes at a specific time point. For the DBG assemblers ABYSS, SOAP2, and VELVET only the peak resident size of the best resulting k-mer parameter are shown and not the summation of all assemblies using different k-mer parameters. (PDF) [file pone.0107014.s009.pdf]

NGA50 length (kb)

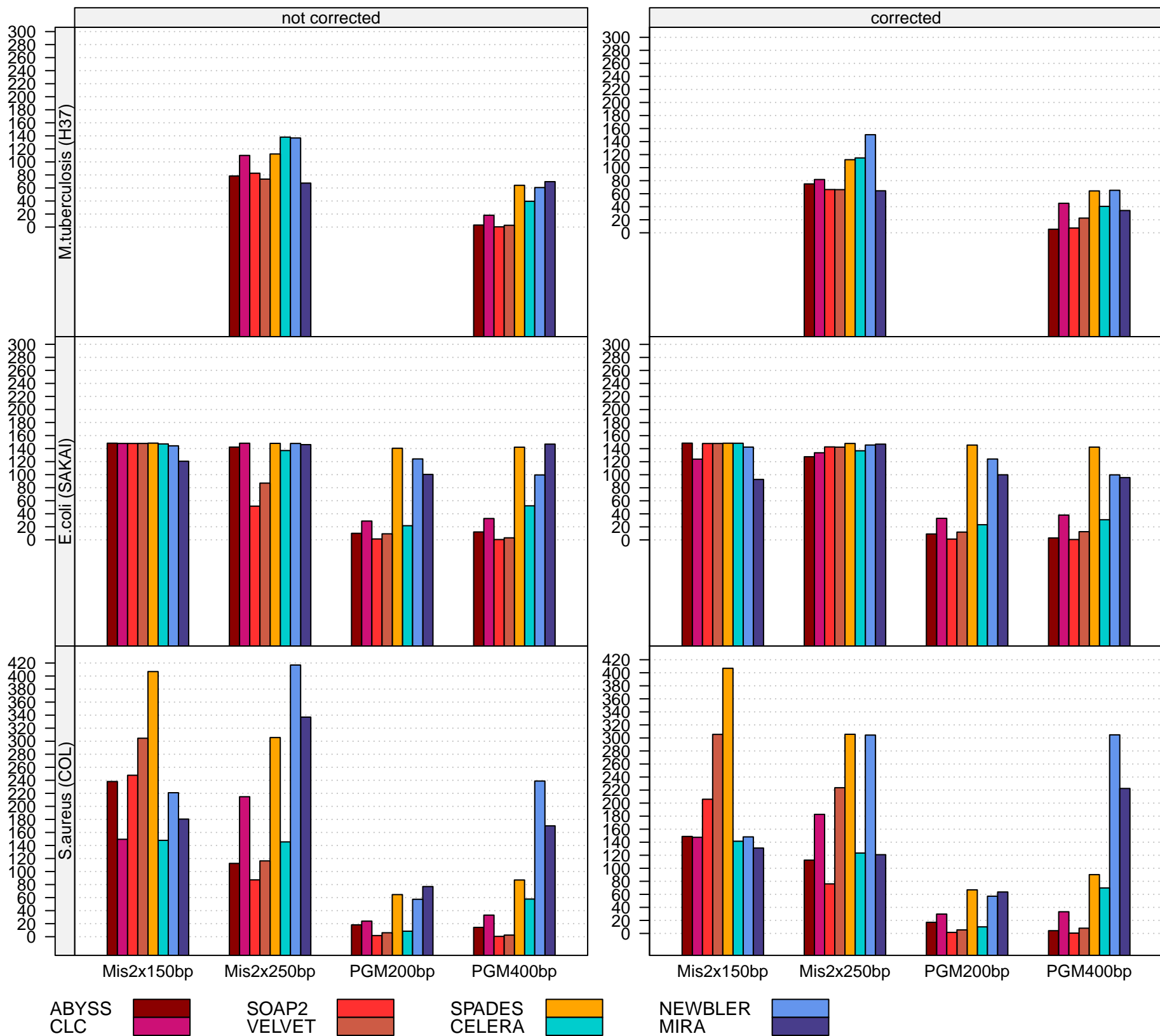

Supplement: Figure S10 — Effect of upstream error correction on de novo genome assemblies. Compared are NGA50 lengths (in kilobase pairs) of assemblies without an upstream read based error correction (left side) with those based on error corrected reads (using BayesHammer on MiSeq data and Coral on PGM data; right side). The NGA50 length is either contig or scaffold based, respectively. Scaffolds for MiSeq 2×150 bp and MiSeq 2×250 bp assemblies obtained by ABYSS, CELERA, CLC, NEWBLER, SOAP2, SPADES, and VELVET; contigs for MiSeq assemblies obtained by MIRA and all PGM assemblies. (PDF) [file pone.0107014.s010.pdf]
