# Supplementary material for: GABenchToB: A Genome Assembly Benchmark Tuned on Bacteria and Benchtop Sequencers
Source: PLoS One. 2014 Sep 8;9(9):e107014. doi: 10.1371/journal.pone.0107014 (PMC4157817; doi:10.1371/journal.pone.0107014)
Supplement: Table S1 — Determined optimal k-mer sizes for DBG assemblies with mandatory k-mer parameterization. (DOC) [file pone.0107014.s011.doc]

**Table S1.** Determined optimal k-mer sizes for DBG assemblies with mandatory k-mer parameterization.

| **Platform** | **Library** | **Strain** | **Optimal k-mer size** | | |
| --- | --- | --- | --- | --- | --- |
|  |  |  | **ABYSS** | **VELVET** | **SOAP2** |
| PGM | 200bp | *E. coli* (Sakai) | 58 | 55 | 127 |
| PGM | 400bp | *E. coli* (Sakai) | 96 | 199 | 127 |
| PGM | 200bp | *S. aureus* (COL) | 38 | 65 | 97 |
| PGM | 400bp | *S. aureus* (COL) | 82 | 187 | 127 |
| PGM | 400bp | *M. tuberculosis* (H37) | 36 | 25 | 127 |
| MiSeq | 2x150bp | *E. coli* (Sakai) | 52 | 75 | 67 |
| MiSeq | 2x250bp | *E. coli* (Sakai) | 100 | 83 | 123 |
| MiSeq | 2x150bp | *S. aureus* (COL) | 80 | 81 | 83 |
| MiSeq | 2x250bp | *S. aureus* (COL) | 110 | 99 | 127 |
| MiSeq | 2x250bp | *M. tuberculosis* (H37) | 76 | 95 | 99 |
