# Supplementary material for: GABenchToB: A Genome Assembly Benchmark Tuned on Bacteria and Benchtop Sequencers
Source: PLoS One. 2014 Sep 8;9(9):e107014. doi: 10.1371/journal.pone.0107014 (PMC4157817; doi:10.1371/journal.pone.0107014)
Supplement: Table S2 — Calculated insert-sizes of MiSeq sequencing libraries. (DOC) [file pone.0107014.s012.doc]

**Table S2.** Calculated insert-sizes of MiSeq sequencing libraries**.**

| **Platform** | **Library** | **Strain** | **Insert-size** | | | |
| --- | --- | --- | --- | --- | --- | --- |
|  |  |  | **Min** | **Max** | **Mean** | **SD** |
| MiSeq | 2x150bp | *E. coli* (Sakai) | 147 | 520 | 329.81 | 105 |
| MiSeq | 2x250bp | *E. coli* (Sakai) | 262 | 539 | 374 | 87 |
| MiSeq | 2x150bp | *S. aureus* (COL) | 142 | 464 | 284.7 | 95.8 |
| MiSeq | 2x250bp | *S. aureus* (COL) | 84 | 480 | 301 | 106 |
| MiSeq | 2x250bp | *M. tuberculosis* (H37) | 293 | 561 | 387 | 86 |
