# Supplementary material for: GABenchToB: A Genome Assembly Benchmark Tuned on Bacteria and Benchtop Sequencers
Source: PLoS One. 2014 Sep 8;9(9):e107014. doi: 10.1371/journal.pone.0107014 (PMC4157817; doi:10.1371/journal.pone.0107014)
Supplement: Text S1 — Supporting Information to GABenchToB: A Genome Assembly Benchmark Tuned on Bacteria and Benchtop Sequencers. (PDF) [file pone.0107014.s013.pdf]

Text S1. Supporting Information to GABenchToB: A Genome Assembly Benchmark Tuned on Bacteria and Benchtop Sequencers .

Genome coverage

The depth of coverage is an important parameter in genome assembly. Both, insufficient as well as extensive coverage can have a negative influence on the assembly outcome. Therefore, finding an optimal target coverage prior to the sequencing effort can help to keep sequencing costs low while still assuring sufficient sequencing data for satisfactory assemblies. Additionally to the main text, in this supplemental text we describe in more detail how altering the depth of coverage affects the assembly outcomes. **Figures S1**, **S2**, and **S3** are showing for the tested assemblers (ABYSS, CELERA, CLC, MIRA, NEWBLER, and SPADES) and all ten data sets the depth of coverage in relation to the NGA50 length, to the number of mis-assemblies, and to the number of assembly errors, respectively. As already pointed out in the main text for *E. coli*, the fact that coverage increases beyond a specific threshold does not necessarily increase assembly contiguity likewise, is in good accordance also with the *S. aureus* and *M. tuberculosis* data (**Figure S1**). Interestingly, the NGA50 lengths are highly fluctuating with altering coverage. The zig-zag pattern of the corresponding curves show clearly how sensitive some assemblers react to coverage changes. A slight coverage increase can have a noticeable positive effect on the assembly whereas another coverage increase by the same amount can completely invalidate this improvement. This is especially apparent for higher coverage ranges as shown by the *S. aureus* data sets. Of course, the non-deterministic behavior of parallelized assembly processes may further contribute to this variation.

These findings are further supported by other metrics. N50 length for the MiSeq and the PGM show comparable saturation and over-saturation effects for most data sets albeit a tendency towards further N50 increases with rising coverage can be observed (data not shown). This can be explained by the observation that sometimes the number of mis-assemblies also increases with rising coverage (**Figure S2**). Most affected here is MIRA, that for some data sets steadily accumulates mis-assemblies, best shown by the PGM data sets above 75-fold coverage. This in turn has a direct opposing effect on NGA50 values explaining the different behavior of N50 vs. NGA50. Whereas the number of assembly errors (mismatches, insertions, and deletions; **Figure S3**) are affected only to a small extent by the depth of coverage. After reaching a plateau at 20-fold to 30-fold coverage, error rates remains relatively stable in most cases.

Nonetheless, it could be argued that the observed variation originates from the randomness in the raw data introduced by the sub-sampling procedure. In order to rule this out, we additionally tested a second sub-sampling approach, i.e. progressive sub-sampling, where each subset is a strict inclusion in all consecutive subsets of a higher coverage. Here, we were interested especially in regions of lower coverages as in those regions the greatest discrepancies between both approaches were to be expected. Therefore, we progressively sub-sampled the *E. coli* and *S. aureus* data sets to subsets of one to a maximum of 100-fold coverage with an increment of 5-fold coverage and compared the consecutive assemblies for NGA50 and number of mis-assemblies (**Figures S4** and **S5**). To sum up, the results are corroborating previous findings: increasing the depth of coverage does not necessarily result in better assemblies. Most interestingly, the curves of the NGA50 plots show the same zig-zag pattern as for the plots based on the random sub-sampling. This clearly emphasizes the sensitivity of the assemblers to coverage changes and refutes the explanation that the variation is only caused by the randomness of the sub-sampling process.

K-mer parametrization of De Bruijn Graph assemblers

As de Bruijn Graph assemblers build upon k-mers of a distinct length, the right k-mer parameterization of such assemblers is vital for the assembly quality. If the k-mer length is chosen too short, the hereupon resulting DBG might suffer from too many prefix-suffix overlaps, i.e. the graph is overly connected. In contrast, a k-mer length close to the read length will, mostly due to sequencing errors, result in graphs with sparse connectivity because of too few overlaps. Both scenarios will lead to suboptimal assemblies and can only be avoided if an optimal k-mer length can be deduced from the input data in advance. This can be performed either by the assembler itself or by specialized third-party software. The naïve alternative, of course, is to try all possible k-mers. In the course of this evaluation, we have followed the naïve approach for all DBG assemblers for which an explicit k-mer parameter specification is mandatory (ABYSS, SOAP2 and VELVET; **Figure S6**). As expected, NGA50 lengths are strongly fluctuating with altering k-mer parameters. Individual best performing k-mers are distributed over a wide scale of possible k-mers and within windows of a certain k-mer range several local NGA50 optima (peaks) can be observed. Best NGA50 lengths for the VELVET assembler ranges between the k-mer parameters k=35 and k=109, for ABYSS between k=56 and k=110, and for SOAP2 between k=63 and k=127 (MiSeq data only). Thereby, no clear correlation of the optimal k-mer parameter, the data set and the assembler can be observed. For instance, highest NGA50 values for the MiSeq 2x250bp *E. coli* data set are achieved with k=98 (ABYSS), k=123 (SOAP2), and k=35 (VELVET). Even within one type of data and one assembler, NGA50 optima differ markedly, e.g. for the ABYSS assembler the three MiSeq2x250bp optima are k=98, k=110, and k=58.

Of the DBG assembler used in this evaluation, the SPADES assembler is a special case. SPADES is initiated with a set of k-mer parameters used to run several consecutive assemblies, which are iteratively incorporated into a final result. For SPADES, the main question with regard to the optimal k-mer parameter set is which individual k-mer should be added to the list of k-mer parameters. Therefore, we used a complete set of all possible k-mers and benchmarked intermediate results of the iterative assembly cycles (**Figure S7A**). Here, two effects can be seen. First, not every additional assembly cycle, i.e. not every additional k-mer parameter, improves the final assembly (e.g., MiSeq 2x250bp S. aureus between the k-mer values of 75 and 97). Secondly, while for the MiSeq data sets the best assemblies were always performed if the set of k-mers ranged to the max. of k=127, PGM assemblies reached their optima always with sets of lower maximum k-mers. Anyway, the assemblies with the full k-mer sets (up to the NGA50 optima) show no improvements over our assemblies

achieved with the default k-mer parameters (**Figure S7B**).

Selection of optimal k-mer parameters

We want to note that we are comparing and evaluating the assemblies using the (genome size based and mis-assembly corrected) NGA50 metric. However, the N50 metric was used to select best resulting k-mer sizes (**Table S1**) for all corresponding assemblies (**Figures 2-3**, **S1-S5** and **S8-S10**) in the consecutive analysis. This is owed to the fact that in *de novo* genome assembly the genome sequence of the organism under consideration is usually unknown. Therefore, neither a correction for genome size nor for mis-assemblies can be performed. Hence, it would be misleading to base the k-mer optimization on NGA50 lengths for a practical evaluation.

De novo assembly receipts

Here, we give detailed information about the specific configuration and parameterization of each assembler used in this study. For all MiSeq data sets, insert-sizes and deduced values were calculated from the data as as described in the methods section of the main text. A corresponding overview of all insert-size metrics is given in **Table S2**. For all hereafter-listed commands, shell variables were used to substitute input dependent variables with appropriate values. Enclosing curly brackets and a leading dollar sign denotes these shell variables. Variable substitutions valid for all commands are the following:

- *THREADS*: Always set to 48, if not stated otherwise.
- *inputFile*: Always refer to the FASTQ file containing the input reads. For MiSeq data sets, this is one single file where both the forward and reverse reads were shuffled pairwise, i.e. interleaved file format. If the assembler prerequisites both files to be given separately, the variable *inputFileA* refers to the FASTQ file containing the forward reads and the variable *inputFileB* to the reverse reads, respectively.
- *prefix*: Always refers to a base naming scheme for all assemblers generated output folders and files. Concrete, this was set to the name of the used assembler, sequencing platform, sequencing library and data set, concatenated by underscores.
- *output*: For assemblers which do not create a distinct output folder, a corresponding folder was created beforehand and assigned to this variable.
- *tmp*: A local temporary directory was used where all input data was transferred to before the assembly execution. This folder also served as the temporary working directory for the duration of the assembly process. However, some assemblers are performing these preparations on their own in which case this variable is used to point to the specific temporary directory location.

As already described, for DBG assemblers with mandatory k-mer parameterization, final assembly evaluations were determined by maximizing N50 lengths. The final k-mer parameter of these main assemblies are listed in **Table S1**.

AbySS

For ABYSS, we used the the following command to start the assemblies:

abyss-pe ${input} k=${i} name=${prefix}_${i} v=-v j=${THREADS} np=${THREADS} mpirun=$

{mpirun}

In dependence on the input data, we set the *input* parameter to '*in=$(inputFile}*' for MiSeq and to '*se=${inputFile}*' for PGM data pointing to the input file locations, respectively. The parameter *k* was set from 32 to 128 and incremented at a step size of two. The *mpirun* parameters points to the location of the Open MPI executable.

Celera

For CELERA, we first generated the specific input format. In the case of MiSeq data with the following command:

fastqToCA -libraryname ${prefix} -technology illumina -type sanger -innie -mates ${inputFile} -insertsize ${mean} ${sd}

In the case of PGM data using this command:

fastqToCA -libraryname ${prefix} -technology 454 -type sanger -reads ${inputFile}

The insert-size parameters *mean* and *sd* were set for each data set to the corresponding means and standard deviations (**Table S2**). Then, a configuration file was created containing the following entries:

cnsConcurrency=24

createACE=1

frgCorrConcurrency=24

frgCorrThreads=2

mbtConcurrency=1

mbtThreads=24

merOverlapperExtendConcurrency=24

merOverlapperSeedConcurrency=24

merOverlapperThreads=2

merylMemory=10240

obtOverlapper=ovl

ovlConcurrency=24

ovlCorrConcurrency=24

ovlHashBits=23

ovlHashBlockLength=200000000

ovlRefBlockSize=200000

ovlOverlapper=ovl

ovlStoreMemory=10240

ovlThreads=2

shell=/usr/bin/env bash

To this configuration file the following entries were added for MiSeq data:

overlapper=ovl

unitigger=bogart

And for PGM data :

overlapper=mer

unitigger=bog

We started the Celera assemblies using the following command:

runCA -d ${output} -p ${prefix} -s ${output}/${prefix}.CELERAconfig ${FRAGINPUT} &> ${output}/ ${prefix}.log

Here, *FRAGINPUT* refers to the input file created using the *fastqToCA* command.

CLC Assembly Cell

To run CLC, we used the following command for MiSeq data:

clc_assembler -o ${output}/${prefix}.result.fas --cpus ${THREADS} -v -p fb ss ${lowInsert} $ {uppInsert} -q ${inputFile} -e ${output}/${prefix}.distanceEstimate.tsv &> ${output}/${prefix}.log

And the following command for PGM data:

clc_assembler -o ${output}/${prefix}.result.fas --cpus ${THREADS} -v -p no -q ${inputFile} &> $ {output}/${prefix}.log

The parameter *lowInsert* and *uppInsert* are referring to the min and max insert-size boundaries (**Table S2**).

GS De Novo Assembler

Before the NEWBLER assembler can be started, a new assembly project has to be created. Therefore, the commands to prepare and start the assemblies for MiSeq data were as follows:

newAssembly -force ${output}
addRun -lib Mates -p ${output} ${inputFile}
runProject -ace -infoall -cpu ${THREADS} -verbose -e ${EXPECT} ${output} &> ${output}/${prefix}.log

Accordingly, for PGM data sets the following commands were used:

newAssembly -force ${output}
addRun -np ${output} ${inputFile}
runProject -ace -infoall -cpu ${THREADS} -verbose -e ${EXPECT} ${output} &> ${output}/${prefix}.log

The *EXPECT* parameter was set to 40 for PGM 200bp data sets and to 75 for all other data sets.

MIRA

In order to start MIRA assemblies, we first generated a manifest configuration file for each assembly. This manifest contains the following entries in the case of MiSeq data:

project = ${prefix}
job = genome,denovo,accurate
parameters = -GE:not=${THREADS} -DI:trt=${tmp} -MI:somrnl=0
readgroup = solexa
technology = solexa
data = ${inputFileA} ${inputFileB}
strain = ${strain}
template_size = ${lowInsert} ${uppInsert}
segment_placement = ---> <---

In the case of PGM data, the manifest files contained the following:

project = ${prefix}

job = genome,denovo,accurate

parameters = -GE:not=${THREADS} -DI:trt=${tmp}

readgroup = iontor

technology = iontor

data = ${inputFile}

strain = ${strain}

Again, the parameters *lowInsert* and *uppInsert* are referring to the min and max insert-size boundaries (**Table S2**). The *strain* parameter was set to the FASTA file containing the complete reference genome sequence of the processed data even though this information was not used during the assembly process. MIRA then was initiated using the following command:

mira ${prefix}.manifest &> ${prefix}.log

SeqMan Ngen

The SEQMAN assembler also operates on configuration files, for which we applied the following general setup:

#!/usr/bin/smng

setDefaultDirectory defaultDirectory:"${output}"

diskPath path:"${tmp}"

project kind: genome_assembly

workflow kind: de_novo

setVectorParam EndCutOff: 25

setVectorParam EndMerMatch: 1

setVectorParam EndRegion: 15

setVectorParam MaxMerGap: 5

setVectorParam MergeTrimGap: 7

setVectorParam MatchSize: 9

setVectorParam MinEndTrimLength: 5

setVectorParam MinMerMatch: 3

setVectorParam MinTrimLength: 30

setRepeatParam MaxMerGap: 10

setRepeatParam MatchSize: 17

setRepeatParam MinEndFlagLen: 25

setRepeatParam MinFlagLength: 50

setRepeatParam MinMerMatch: 2

setContaminantParam MatchSize: 17

setContaminantParam MinMerMatch: 12

setQualityParam EndRegion: 5

setQualityParam MaxN: 2

setQualityParam MaxNHiQual: 1

setQualityParam MinAveHiQual: 22

setQualityParam MinAveLowQual: 20

setQualityParam MinEndBaseQual: 15

setQualityParam NTrimWinLength: 7

setQualityParam WinLength: ${WINLENGTH}

${LOADSEQ}

setParam AllowConstraintBased: true

setParam FixedCoverage: 20

setParam CoverageType: genome

setParam DefaultQuality: 15

setParam GapPenalty: 30

setParam GenomeLength: 5000000

setParam HaploidSNP: false

setParam HaploidThreshold: 0

setParam LowCoverageThreshold: 0

setParam MatchScore: 10

setParam MatchWindowLength: 50

setParam MaxContigs: 0

setParam MatchRepeatPercent: 150

setParam MaxUsableCount: 25

setParam Minimizer: 1

setParam MismatchPenalty: 20

setParam SkipRealign: false

setParam SNPMatchPercentage: 90

setParam SNPPasses: 2

setParam SplitFalseJoins: false

setParam SplitTemplateContigs: false

setParam TemplateDefaultQuality: 500

setParam UseRepeatHandling: true

${MATCHING}

assemble trimEnds: true vectScan: false repeatScan: false contamScan: false doAssemble: true

${REMOVE}

WriteUnassembledSeqs file:"${output}/${prefix}.unasm.fas" saveTrimmed:false

saveProject file:"${output}/${prefix}.ace" format:Phrap

saveProject file:"${output}/${prefix}.fas" format:Fasta

saveReport file:"${output}/${prefix}.info"

setAssemblyReport file:"${output}/${prefix}.report" name:"${prefix}"

closeProject

In dependence of the input, SEQMAN specific variables were replaced as follows. For MiSeq data:

WINLENGTH := '5'

LOADSEQ :=

'l*oadSeq*

file:"${inputFile}"

multiplex:false

groupName:"${prefix}"

seqTech:"normalScore"

mergePairs:false

isPair:paired

SetPairSpecifier pairs:{ {forward: "(.)/1" reverse:"(.*)/2" min: ${lowInsert} max: ${uppInsert}*

} }'

MATCHING :=

'*setParam MaxGap: 6*

setParam MatchSize: 21

setParam MatchSpacing: 50

setParam MinMatchPercent: 93

setParam TrimToMer: false'

REMOVE := '*removeSmallContigs minSeqs: 100 minLength: 0*'

For PGM data sets, the following replacement took place:

WINLENGTH := '*10*'

LOADSEQ :=

'*loadSeq*

file:"${inputFile}"

multiplex:false

groupName:"${prefix}"

seqTech:"IonTorrent"

mergePairs:false'

MATCHING :=

'*setParam MaxGap: 15*

setParam MatchSize: 19

setParam MatchSpacing: 20

setParam MinMatchPercent: 90

setParam TrimToMer: true'

REMOVE := '*removeSmallContigs minSeqs: 10 minLength: 0*'

The assemblies were then started using this command:

smng ${output}/${prefix}.SEQMANconfig &> ${output}/${prefix}.log

SOAPdenovo2

The SOAP2 assembler was used in two different compiled binary versions with regard to the full

possible k-mer range. For k-mer parameters up to 63 the pre-compiled 63mer version of the SOAPdenovo2-bin-LINUX-generic-r238 release were used and for k-mers from 65 to 127 the 127mer version, respectively. The following command line was used to initiate the assemblies:

SOAPdenovo-${kmerVersion}mer all -p ${THREADS} -s ${output}/${prefix}.SOAP2config -o ${output}/${prefix}_${i} -K ${i} &> ${output}/${prefix}_${i}.log

The k-mer variable was set to values between 13 and 127 incremented with a step size of two. The configuration file containing the following parameters for PGM data sets:

max_rd_len=999
[LIB]
asm_flags=3
rank=1
q=${inputFile}

For MiSeq data sets, the configuration file contained following entries:

max_rd_len=999
[LIB]
avg_ins=${mean}
reverse_seq=0
asm_flags=3
rank=1
q1=${inputFileA}
q2=${inputFileB}

The *mean* parameters were set to the mean insert-size as listed in **Table S2**.

SPAdes

We initiated SPADES using the following configuration for MiSeq data:

spades.py --12 ${inputFile} --careful -o ${output}/${prefix} -t $THREADS --tmp-dir ${tmp} -k 21,33,55,77,99,127 &> ${prefix}.log

And for PGM data using the following command:

spades.py -s ${inputFile} --only-assembler -o ${output}/${prefix} -t $THREADS --tmp-dir ${tmp} -k 21,33,55,77,99 &> ${prefix}.log

Although for SPADES a default comma-separated list of k-mer sizes is defined (21,33,55), we used these different set of parameters for the MiSeq and the PGM data sets as suggested by the SPADES authors (personal communication). Also, as described in the main text, we tested SPAdes using increasing lists with increments of k-mer sizes but due to it's unique assembly technique the k-mer optimization problem does not really apply here and brute force parameter optimization is practically intractable.

Velvet

For the VELVET assemblies we used five different binary versions. Here, velvet was compiled five times with altering the Makefile parameter MAXKMERLENGT to 31, 83, 127, 177, and 227, respectively. The binaries were differentially named by adding an according suffix. In dependence of the actual processed k-mer parameter of the velvet command the velvet binary with minimal MAXKMERLENGT was used for the assembly, e.g. for k-mers below 32 velvet configured with MAXKMERLENGT=31 was used, and so forth. We initiated the Velvet assemblies using the below listed command lines.

MiSeq data:

velveth.${kmerVersion} ${output}/${prefix}_${i} ${i} -shortPaired -fastq ${inputFile} &> ${output}/${prefix}_${i}.velveth.log

velvetg.${kmerVersion} ${output}/${prefix}_${i} -cov_cutoff auto -exp_cov auto -exportFiltered yes -amos_file yes -ins_length ${size} -ins_length_sd ${sd} &> ${output}/${prefix}_${i}.velvetg.log

PGM data:

velveth.${kmerVersion} ${output}/${prefix}_${i} ${i} -short -fastq ${inputFile} &> ${output}/${prefix}_${i}.velveth.log

velvetg.${kmerVersion} ${output}/${prefix}_${i} -cov_cutoff auto -exp_cov auto -exportFiltered yes -amos_file yes &> ${output}/${prefix}_${i}.velvetg.log

The k-mer parameter *${i}* was set to values of the range between 13 and 227 with an increment of two. To enable parallelization, velvet was additionally compiled with OPENMP enabled and the following environment variables were exported to the executing shell prior to the assemblies:

export OMP_NUM_THREADS=${THREADS}-1

export OMP_THREAD_LIMIT=${THREADS}
