# Supplementary figures and images for: Correction: GABenchToB: A Genome Assembly Benchmark Tuned on Bacteria and Benchtop Sequencers
Source: PLoS One. 2015 Mar 19;10(3):e0118741. doi: 10.1371/journal.pone.0118741 (PMC4366415; doi:10.1371/journal.pone.0118741)

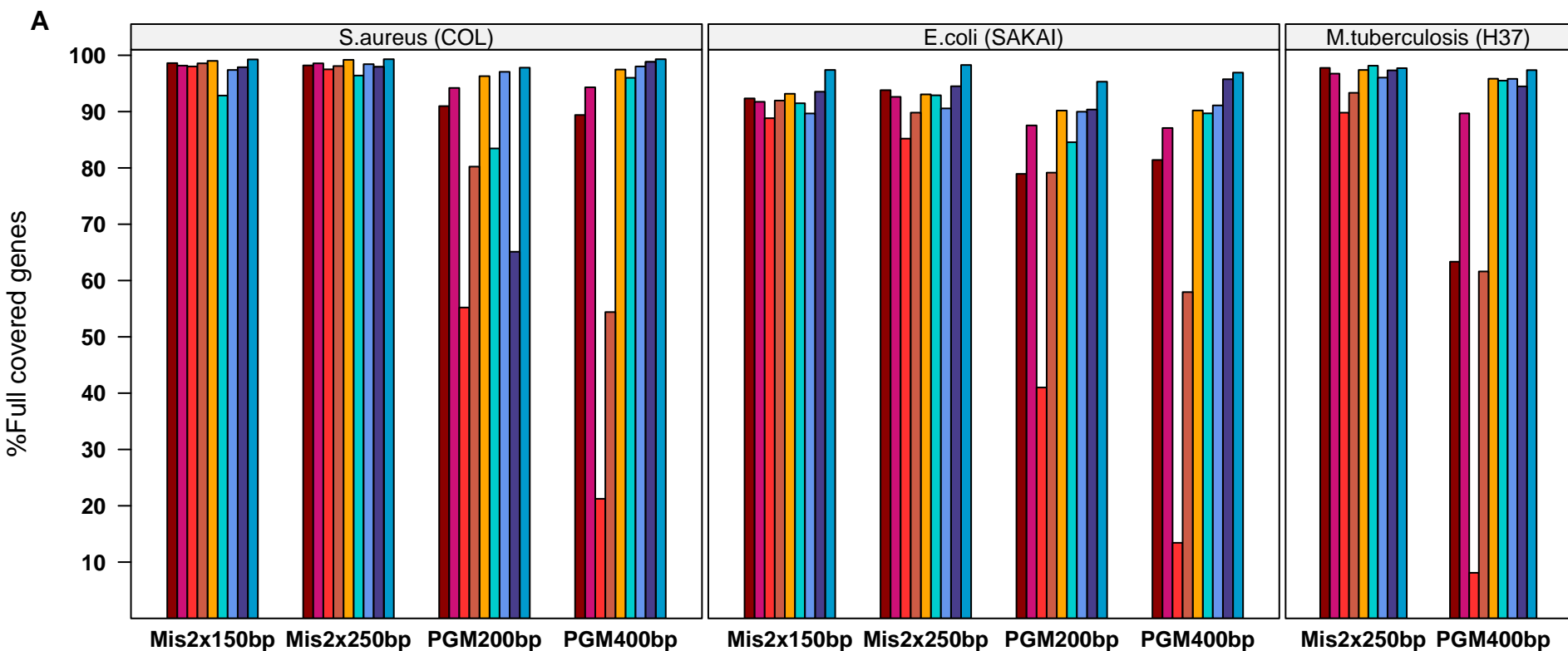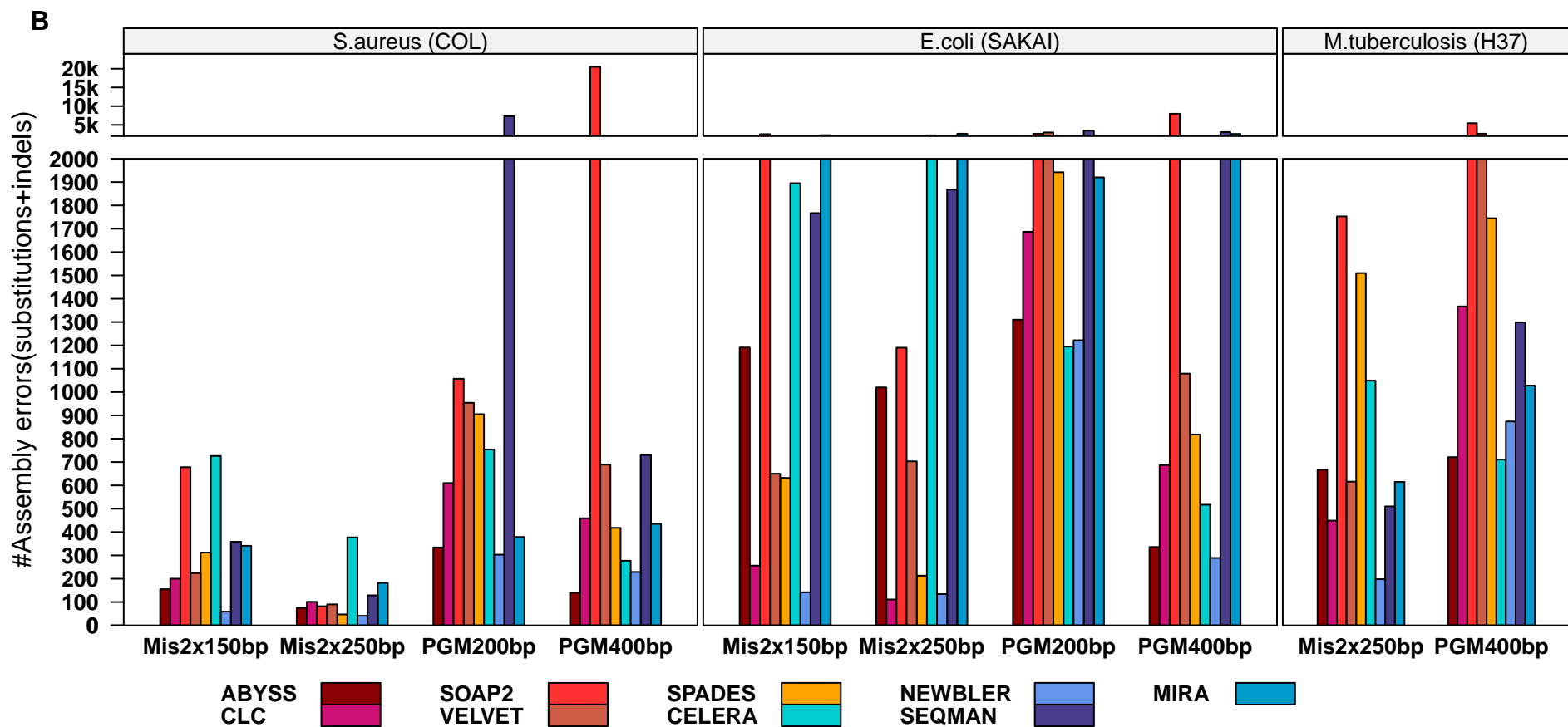

Supplement: S8 Figure — Based on the percentage of full covered genes (A) and the number of assembly errors (B, combining substitutions, insertions, and deletions). Full covered genes are completely covered positions in the reference genome where a gene annotation was provided (based on all chromosomal and plasmid genes). The numbers of assembly errors are either contig or scaffold based, respectively. Scaffolds for MiSeq 2×150 bp and MiSeq 2×250 bp assemblies obtained by ABYSS, CELERA, CLC, NEWBLER, SOAP2, SPADES, and VELVET; contigs for MiSeq assemblies obtained by MIRA and SEQMAN as well as for all PGM assemblies. doi:10.1371/journal.pone.0107014.s008 (PDF) [file pone.0118741.s001.pdf]
